# KRAS mutants confer platinum resistance by regulating ALKBH5 posttranslational modifications in lung cancer

**DOI:** 10.1172/JCI185149

**Published:** 2025-02-04

**Authors:** Fang Yu, Shikan Zheng, Chunjie Yu, Sanhui Gao, Zuqi Shen, Rukiye Nar, Zhexin Liu, Shuang Huang, Lizi Wu, Tongjun Gu, Zhijian Qian

**Affiliations:** 1 Department of Medicine, University of Florida Health Cancer Center and; 2 Department of Biochemistry and Molecular Biology, University of Florida, Gainesville, Florida, USA.; 3 Versiti Blood Research Institute, Milwaukee, Wisconsin, USA.; 4 Department of Anatomy & Cell Biology, University of Florida, Gainesville, Florida, USA.; 5 Department of Molecular Genetics and Microbiology, University of Florida Health Cancer Center, University of Florida Genetic Institute, University of Florida, Gainesville, Florida, USA.; 6 Department of Biostatistics, University of Florida, Gainesville, Florida, USA.

**Keywords:** Cell biology, Oncology, Epigenetics, Lung cancer, Signal transduction

## Abstract

Constitutively active mutations of *KRAS* are prevalent in non–small cell lung cancer (NSCLC). However, the relationship between these mutations and resistance to platinum-based chemotherapy and the underlying mechanisms remain elusive. In this study, we demonstrate that *KRAS* mutants confer resistance to platinum in NSCLC. Mechanistically, KRAS mutants mediate platinum resistance in NSCLC cells by activating ERK/JNK signaling, which inhibits AlkB homolog 5 (ALKBH5) *N*^6^-methyladenosine (m^6^A) demethylase activity by regulating posttranslational modifications (PTMs) of ALKBH5. Consequently, the KRAS mutant leads to a global increase in m^6^A methylation of mRNAs, particularly damage-specific DNA-binding protein 2 (*DDB2*) and *XPC*, which are essential for nucleotide excision repair. This methylation stabilized the mRNA of these 2 genes, thus enhancing NSCLC cells’ capability to repair platinum-induced DNA damage and avoid apoptosis, thereby contributing to drug resistance. Furthermore, blocking KRAS-mutant–induced m^6^A methylation, either by overexpressing a SUMOylation-deficient mutant of ALKBH5 or by inhibiting methyltransferase-like 3 (METTL3) pharmacologically, significantly sensitizes *KRAS*-mutant NSCLC cells to platinum drugs in vitro and in vivo. Collectively, our study uncovers a mechanism that mediates KRAS-mutant–induced chemoresistance in NSCLC cells by activating DNA repair through the modulation of the ERK/JNK/ALKBH5 PTM-induced m^6^A modification in DNA damage repair–related genes.

## Abstract 2

KRAS mutants drive platinum resistance in lung cancer cells by regulating DNA damage repair-related genes in an m6A-dependent manner

## Graphical abstract

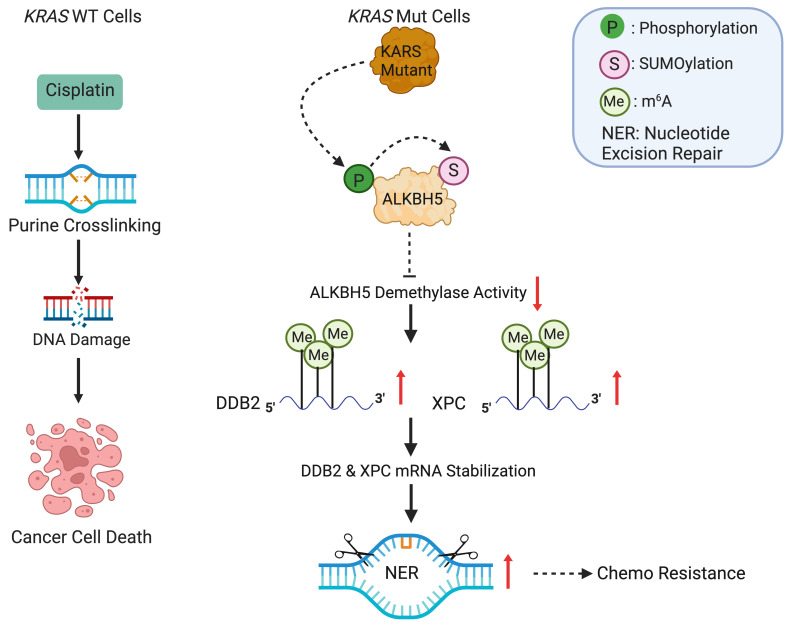

## Introduction

Non–small cell lung cancer (NSCLC) is a frequently diagnosed malignancy and a leading cause of cancer-related deaths worldwide (1). Even when patients with NSCLC receive a combination of surgery and chemotherapy, the survival rate remains low due to cancer cell metastasis, invasion, and drug resistance (2). Consequently, there is an urgent need to identify effective targets for inhibiting drug resistance in NSCLC.

Mutations in *KRAS* have been detected in up to 25% of cases of NSCLC, which accounts for 85% of all lung cancer cases (3, 4). Although *KRAS* has been recognized as one of the most frequently mutated oncogenes in human malignancies since 1969, the lack of druggable pockets on the KRAS protein surface has resulted in only 2 FDA-approved drugs until now (5, 6). However, these 2 FDA-approved KRAS inhibitors only specifically target a particular KRAS mutation (KRAS G12C) (6, 7). Currently, platinum-based analogs such as cisplatin and carboplatin are still commonly used for patients with KRAS-mutant NSCLC. Nonetheless, effectiveness of chemotherapy in *KRAS*-mutant NSCLC patients has been limited, failing to produce a lasting response (1, 8). Reports have indicated that *KRAS*-mutant NSCLC patients responded less favorably to cytotoxic therapy compared with patients with WT *EGFR* and *KRAS* genes (9–11). However, the question of whether and how *KRAS* mutations confer NSCLC platinum resistance remains unresolved.

Despite more than 170 chemical modifications on RNAs having been identified to date, *N*^6^-methyladenosine (m^6^A) methylation remains the most abundant internal modification on eukaryotic mRNA (12). m^6^A methylation can be dynamically regulated by m^6^A writers methyltransferase-like 3 (METTL3) and METTL14 as well as m^6^A erasers fat mass– and obesity-associated protein (FTO) and AlkB homolog 5 (ALKBH5) (13–18). This reversible m^6^A methylation constitutes a new layer of posttranscriptional regulation of gene expression. m^6^A plays a pivotal role in governing almost all aspects of RNA metabolism, encompassing splicing, localization, translation, and stability, by recruiting a group of proteins termed as m^6^A readers. Although numerous studies have suggested that m^6^A methylation plays crucial roles in the occurrence and development of various cancer types, including NSCLC, the role of m^6^A methylation in chemoresistance in *KRAS*-mutant NSCLC remains elusive (19–21).

In this study, we investigated the role of *KRAS* constitutively active mutations in conferring platinum resistance in NSCLC. We demonstrate that KRAS mutants induce chemoresistance in NSCLC by amplifying EKR/JNK signaling-mediated ALKBH5 posttranslational modifications (PTMs), including phosphorylation and SUMOylation. ALKBH5 PTMs lead to inhibition of ALKBH5 demethylase activity, resulting in an upregulation of m^6^A methylation within over a hundred transcripts with alteration of expression. Among these transcripts, damage-specific DNA-binding protein 2 (DDB2) and XPC, which play an essential role in nucleotide excision (22, 23), are significantly upregulated as a consequence of an increase in m^6^A methylation in these transcripts. Notably, blocking the KRAS mutation–induced m^6^A increase in the DDB2 and XPC transcripts by METTL3 inhibition substantially sensitizes NSCLC cells to platinum treatment, both in vitro and in vivo. This discovery provides a promising new avenue for the treatment of KRAS-mutant NSCLC. Collectively, our results illustrate how mRNA m^6^A modification adds an additional layer of complexity in mediating KRAS mutation–induced platinum resistance in NSCLC by regulating the expression of genes involved in DNA damage response. This study also represents the instance of a mutant *KRAS* oncogene hijacking the ALKBH5 PTMs/m^6^A methylation–mediated DNA damage response pathway to confer resistance to cytotoxic drugs in lung cancer cells.

## Results

### KRAS constitutively active mutations are associated with NSCLC platinum resistance.

**Figure 1:**
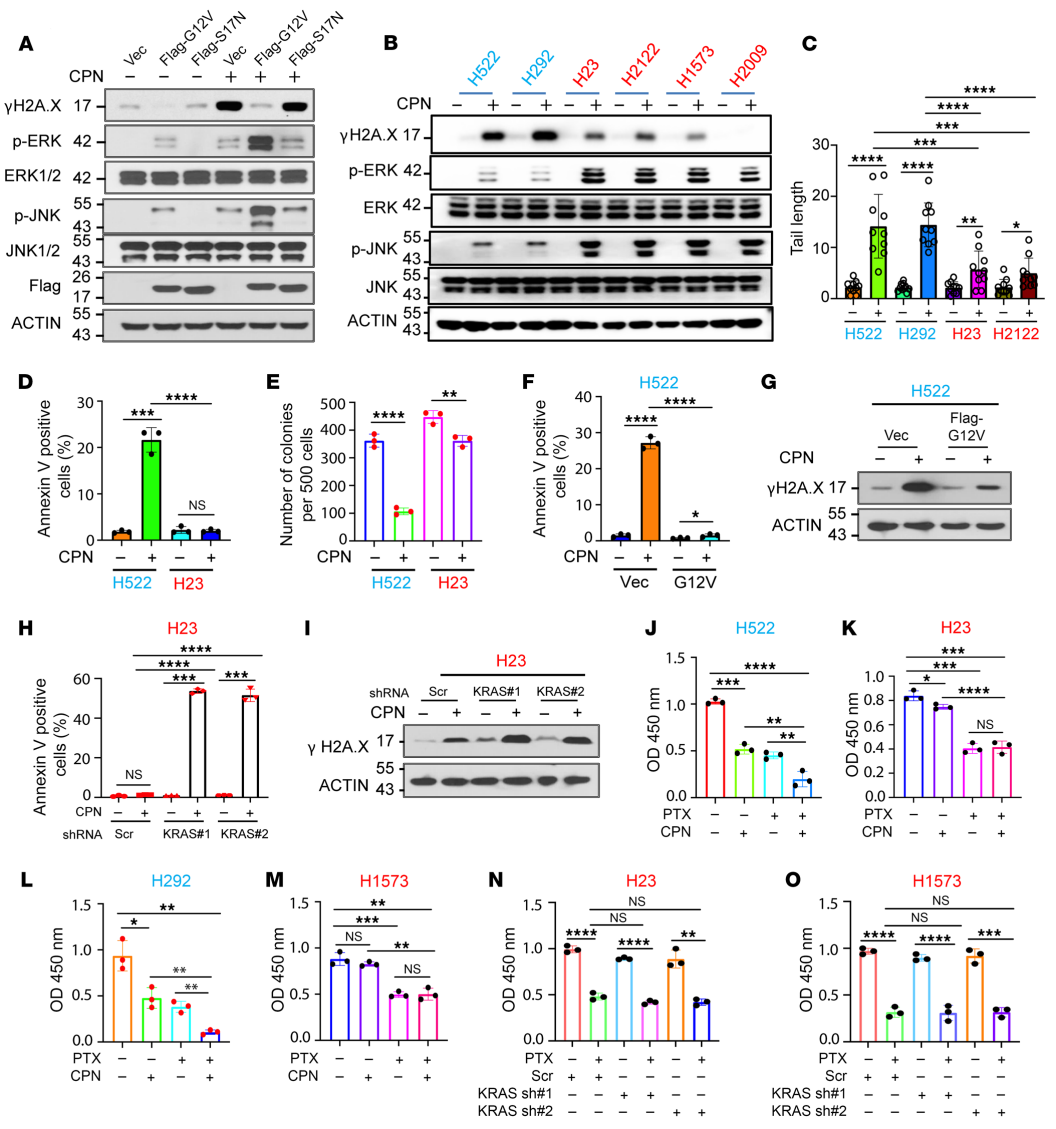
*KRAS* constitutively active mutation confers NSCLC platinum resistance. (**A**) Western blot analysis showing protein levels as indicated in BEAS-2B cells. (**B**) Western blot analysis showing the protein levels as indicated in *KRAS* WT or -mutant NSCLC cells with or without cisplatin treatment. (**C**) Comet analysis for *KRAS* WT and mutant NSCLC cells with or without cisplatin treatment. (**D**) Cell apoptosis analyses for *KRAS* WT or -mutant NSCLC cells with or without cisplatin treatment. (**E**) Colony-forming analyses for *KRAS* WT or mutant NSCLC cells with or without cisplatin treatment. (**F**) Annexin V staining analysis showing that overexpression of the KRAS-mutant significantly inhibits cisplatin-induced cell apoptosis in NCI-H522 cells. (**G**) Western blot analysis showing that overexpression of the KRAS-mutant significantly inhibits cisplatin-induced DNA damage in NCI-H522 cells. (**H**) Annexin V staining analysis showing that *KRAS* KD significantly facilitates cisplatin-induced cell apoptosis in NCI-H23 cells. (**I**) Western blot analysis showing that *KRAS* KD significantly promotes cisplatin-induced DNA damage in NCI-H23 cells. (**J**–**M**) CCK8 analyses showing the effect of cisplatin and PTX treatment on the cell proliferation of *KRAS* WT and -mutant NSCLC cells. (**N** and **O**) CCK8 analysis indicating the effect of *KRAS* KD on PTX sensitivity of KRAS-mutant cells. In **C**–**F**, **H**, and **J**–**O**, data are presented as mean ± SD, with ordinary 1-way ANOVA with Dunnett’s multiple-comparison test used for **C**, **D**, **F**, **H**, and **J**–**O** and 2-tailed Student’s *t* test for **E**. *P < 0.05; **P < 0.01; ***P < 0.001; ****P < 0.0001.

Despite the widespread occurrence of KRAS constitutively active mutations in lung cancers (24–26), the association between these mutations and platinum resistance in NSCLC has not been fully investigated. KRAS G12C (41%), KRAS G12V (22%), KRAS G12D (12%), and KRAS G12A (9.3%) represent the most commonly observed mutations in KRAS within lung cancers (7, 27). We first established BEAS-2B cells derived from normal bronchial epithelium, stably expressing vector, KRAS constitutively active form (KRAS G12V), or a KRAS enzymatic mutant (KRAS S17N) and treated these cells with either DMSO or cisplatin. As shown in Figure 1A, the overexpression of constitutively active KRAS (KRAS G12V) but not KRAS enzymatic mutant (KRAS S17N) led to an increase in phosphorylated ERK and JNK protein levels in BEAS-2B cells. Notably, cisplatin treatment activates ERK/JNK signaling, and this activation can be further enhanced by the overexpression of KRAS G12V (Figure 1A). Meanwhile, cisplatin exposure significantly induced DNA damage in BEAS-2B cells, as evidenced by an increased expression of phosphorylated γH_2_AX, a sensitive marker of DNA damage (Figure 1A). Strikingly, KRAS-G12V significantly bolstered the resistance of BEAS-2B cells to cisplatin-induced DNA damage (Figure 1A). Next, we treated KRAS WT NSCLC cells, including NCI-H522 and NCI-H292, KRAS G12C-mutant NSCLC cells, such as NCI-H23 and NCI-H2122, and KRAS G12A-mutant NSCLC cells, such as NCI-H1573 and NCI-H2009, with either DMSO or cisplatin. Consistently, ERK/JNK signaling was more significantly activated, resulting in lower DNA damage in response to the chemotherapeutic drug in KRAS-mutant NSCLC cell lines, including NCI-H23, NCI-H2122, NCI-H1573, and NCI-H2009 as compared with KRAS WT lung cancer cell lines such as NCI-H522 and NCI-H292 (Figure 1B). Precise single-cell DNA damage analysis using the alkaline comet assay revealed that KRAS WT NSCLC cells exhibit greater sensitivity to cisplatin-induced DNA damage compared with KRAS mutant lung cancer cells (Figure 1C and Supplemental Figure 1A; supplemental material available online with this article; https://doi.org/10.1172/JCI185149DS1). Additionally, cisplatin treatment markedly induced apoptosis in NCI-H522, whereas it had a marginal effect on apoptosis of NCI-H23 cells (Figure 1D and Supplemental Figure 1B). We next examined colony forming ability of these cells. As shown in Figure 1E and Supplemental Figure 1C, NCI-H522 (KRAS WT) gave rise to fewer colonies than NCI-H23 (KRAS G12C) when the cells were treated with cisplatin. Collectively, these results suggest a positive correlation between KRAS constitutively active mutations and platinum resistance in NSCLC cells.

### KRAS constitutively active mutations confer NSCLC platinum resistance.

To rigorously investigate whether KRAS mutations confer platinum resistance in lung cancer cells, we adopted 2 approaches: overexpressing a constitutively active KRAS mutant in NCI-H522 (KRAS WT) and knocking down KRAS in NCI-H23 (KRAS G12C) cells. KRAS G12V overexpression markedly inhibited cisplatin-induced DNA damage and cell apoptosis in NCI-H522 cells (Figure 1, F and G, and Supplemental Figure 1D). Conversely, KRAS knockdown (KD) greatly enhanced cisplatin-induced DNA damage and cell apoptosis in NCI-H23 cells (Figure 1, H and I, and Supplemental Figure 1, E and F). In addition to the platinum-based drugs, paclitaxel (PTX) is also a frequently used chemotherapeutic drug in lung cancer treatment (28–31). Therefore, we next examined whether KRAS mutants induce PTX resistance in lung cancer cells. As shown in Figure 1, J–O, and Supplemental Figure 1G, KRAS-mutant NSCLC cells including H23 and H1573 and KRAS-WT NSCLCs including H522 and H292 are responsive to PTX treatment while *KRAS* KD did not increase the sensitivity of H23 and H1573 NSCLC cells to PTX treatment. However, we also observed that ERK/JNK signaling is highly activated in KRAS-mutant cells, exhibiting lower levels of DNA damage compared with KRAS WT cells when treated with other DNA damage reagents, such as doxorubicin and etoposide (Supplemental Figure 2, A and B). Taken together, these results provide compelling evidence that KRAS constitutively active mutations specifically confer platinum resistance, as well as other DNA damage inducers, but not PTX in NSCLC cells.

### KRAS-mutant-induced NSCLC platinum resistance is not mediated by ABC transporters.

ATP-binding cassette (ABC) transporters are the largest and oldest membrane proteins in humans, which pump out various toxic compounds from the cells. The major cause of multidrug resistance (MDR) and chemotherapeutic failure is believed to be the efflux of toxic drugs mediated by ABC transporters (32–34). Therefore, we next examined whether KRAS mutant-mediated platinum resistance is possibly mediated by ABC transporters. As shown in Supplemental Figure 2, C–E, the expression of ABC transporters including ABCB1, ABCG2, and ABCC1 is comparable in KRAS-WT and mutant NSCLC cells. Additionally, KRAS KD did not affect the expression of ABC transporters in KRAS-mutant lung cancer cells (Supplemental Figure 2, F–H). Together, these data suggest that KRAS-mutant–mediated NSCLC platinum resistance is not attributed to the dysregulation of ABC transporters.

### The KRAS mutant regulates global mRNA m^6^A methylation via controlling ALKBH5 phosphorylation and SUMOylation.

**Figure 2:**
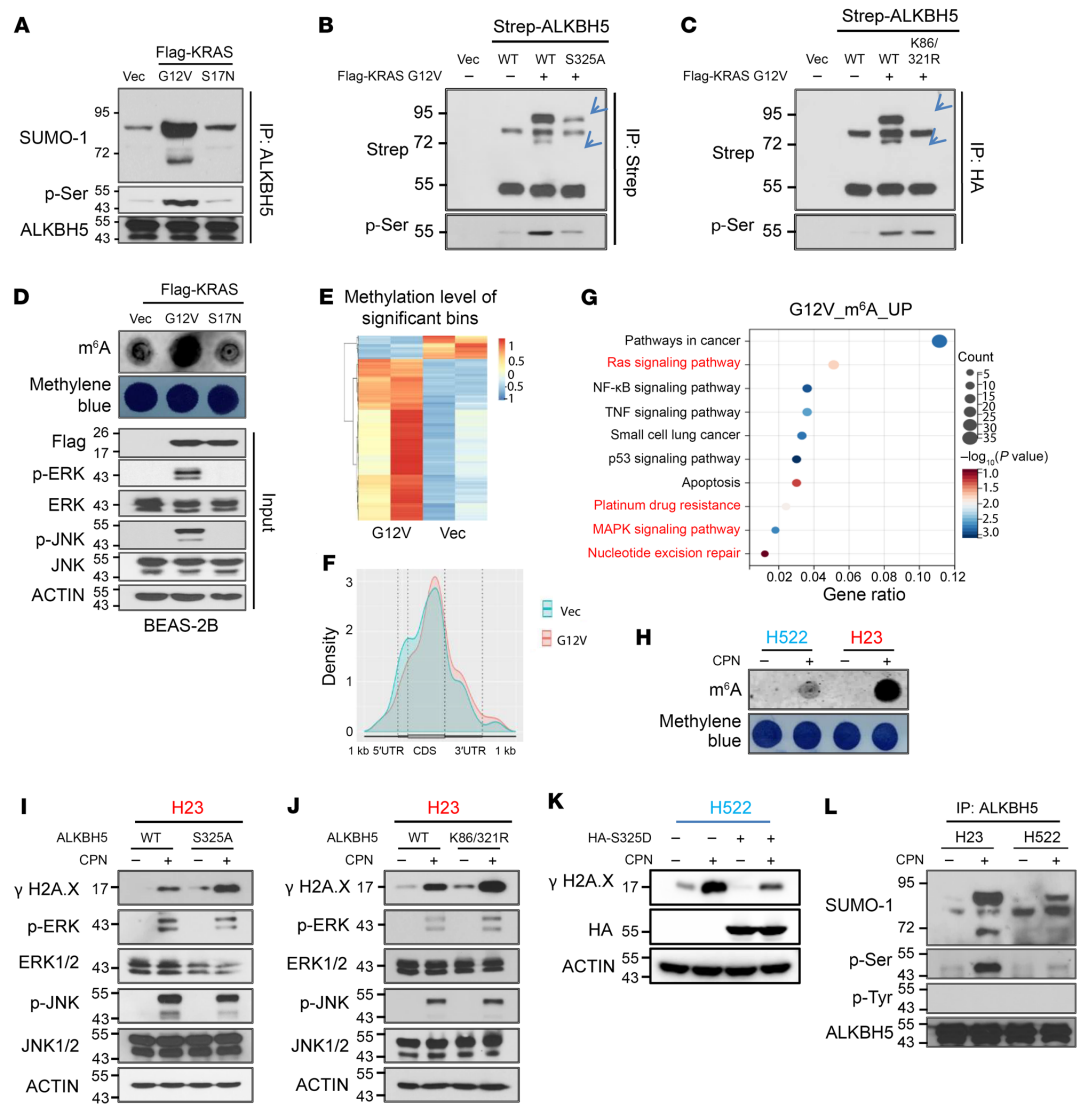
The constitutively active KRAS mutant regulates global mRNA m^6^A methylation via controlling ALKBH5 phosphorylation and SUMOylation. (**A**) Denaturing IP analysis suggests overexpression of the constitutively active KRAS mutant significantly induces ALKBH5 phosphorylation and SUMOylation in BEAS-2B cells. (**B**) IP analysis suggesting the KRAS-mutant mediates ALKBH5 phosphorylation at serine residue 325. (**C**) Denaturing IP analysis suggests that overexpression of the constitutively active KRAS-mutant induces ALKBH5 SUMOylation at lysine residues 86 and 321. (**D**) Dot-blot analysis suggests global mRNA m^6^A methylation could be induced by overexpression of the constitutively active KRAS-mutant. (**E**) Heatmap showing mRNA transcripts with significant m^6^A modification alterations upon KRAS G12V overexpression in NCI-H522 cells identified by m^6^A-seq analysis. (**F**) The frequency distribution of m^6^A peaks across the length of mRNA transcripts shown by metagene analysis in empty vector and KRAS G12V–overexpressed NCI-H522 cells. (**G**) GO analysis of genes, of which m^6^A methylation was significantly upregulated by KRAS G12V overexpression. (**H**) Dot-blot analysis indicating global mRNA m^6^A methylation in *KRAS* WT and -mutant NSCLC cells with or without cisplatin treatment. (**I**) Western blot analysis suggests that overexpression of the ALKBH5 phosphorylation-deficient mutant significantly sensitizes *KRAS*-mutant harboring NCI-H23 cells to cisplatin-induced DNA damage. (**J**) Western blot analysis suggests that overexpression of the ALKBH5 SUMOylation-deficient mutant significantly sensitizes *KRAS*-mutant harboring NCI-H23 cells to cisplatin-induced DNA damage. (**K**) Western blot analysis indicates that overexpression of the phosphorylation-mimic mutant ALKBH5 S325D significantly enhances the cisplatin sensitivity of *KRAS* WT H522 cells. (**L**) Denaturing IP analysis showing ALKBH5 phosphorylation and SUMOylation in *KRAS* WT and -mutant NSCLC cells with or without cisplatin treatment.

Our previously published study demonstrated that mammalian cells activate ERK/JNK signaling to induce m^6^A methylation in DNA repair-related genes. This process safeguards the genomic stability by regulating ALKBH5 PTMs in response to oxidative stress (35). The ERK/JNK signaling pathway can be activated by ROS stress and oncogenes such as *KRAS* (24, 36–38). To examine whether the KRAS mutant regulates PTMs of ALKBH5, we established BEAS-2B cells, stably expressing vector, constitutively active KRAS mutant (KRAS G12V), and KRAS enzymatic mutant (KRAS S17N). Denaturing immunoprecipitation (IP) analysis of ALKBH5 revealed that expression of constitutively active KRAS significantly induced endogenous ALKBH5 phosphorylation and SUMOylation (Figure 2A and Supplemental Figure 3A). Consistently, inhibition of KRAS G12C by sotorasib, or ERK by PD0325901, markedly reduced both phosphorylation and SUMOylation of ALKBH5 in NCI-H23 cells. These findings suggest that ALKBH5 PTMs, including phosphorylation and SUMOylation, are driven by KRAS/ERK signaling (Supplemental Figure 3, B and C). In addition, both ALKBH5 phosphorylation-deficient mutant S325A and ALKBH5 SUMOylation-deficient mutant ALKBH5 K86R/K321R significantly reduced KRAS G12V–induced ALKBH5 phosphorylation and SUMOylation (Figure 2, B and C, and Supplemental Figure 3, D and E). These findings suggest that the constitutively active KRAS mutant induces ALKBH5 phosphorylation at serine 325 (S^325^), and SUMOylation at lysines 86 (K^86^) and 321 (K^321^). Our previous study suggests that ALKBH5 phosphorylation triggers its SUMOylation, which in turn inhibits its m^6^A demethylase activity (35). Therefore, we checked whether the constitutively active KRAS mutant induces global mRNA m^6^A modification. Consistently, ectopic expression of KRAS G12V but not KRAS S17N markedly increased global mRNA m^6^A methylation in BEAS-2B cells (Figure 2D). To further determine the effect of the KRAS constitutively mutant on mRNA m^6^A methylation transcriptome wide, we performed m^6^A-Seq analyses. We observed that KRAS G12V overexpression led to 1,542 m^6^A peak alterations in total among transcripts (log_2_ fold change [log_2_FC] > 0.3 or log_2_FC< –0.3, *P* < 0.05). Consistent with previous studies (13, 39, 40), the identified m^6^A peaks are located in sequences containing the canonical m^6^A methylation consensus motif RRACH (R = G or A; H = A, C, or U; where A is converted to m^6^A) (Supplemental Figure 3F). In line with the m^6^A level determined by dot blot, m^6^A-Seq results revealed that the majority of m^6^A peaks are upregulated upon KRAS G12V expression. Overall, 1,259 peaks were upregulated and 283 peaks were downregulated (Figure 2, E and F, and Supplemental Figure 3G). Additionally, Gene Ontology (GO) analysis of 1,259 m^6^A peaks that were significantly upregulated upon KRAS G12V overexpression showed that these peaks are enriched in the genes involved in pathways including RAS and MAPK signaling pathways, platinum drug resistance, and nucleotide excision repair (NER) (Figure 2G). Platinum-based drugs serve as antitumor drugs mainly by facilitating cancer cells DNA damage through inducing crosslink formation between purine nucleotides (22, 41, 42). m^6^A-seq analysis suggests that the constitutively active KRAS mutant overexpression led to an m^6^A increase in the genes associated with NER, suggesting an important role of the NER pathway in KRAS-mutant–mediated platinum resistance in lung cancer cells. Consistently, cisplatin-induced m^6^A increase in KRAS-mutant NCI-H23 cells was significantly higher as compared with KRAS WT NCI-H522 cells (Figure 2H). Meanwhile, inhibition of KRAS G12C or ERK effectively blocked cisplatin-induced m^6^A methylation in KRAS G12C-mutant NCI-H23 cells, suggesting that activation of KRAS/ERK signaling is responsible for the increased m^6^A methylation observed following cisplatin treatment (Supplemental Figure 3, H and I). Additionally, blocking mRNA m^6^A increase by expression of either ALKBH5 S325A or ALKBH5 K86/321R significantly sensitized NCI-H23 cells to cisplatin-induced DNA damage (Figure 2, I and J). Conversely, overexpression of the ALKBH5 phosphorylation-mimic mutant ALKBH5 S325D in KRAS WT H522 cells significantly increased their resistance to cisplatin (Figure 2K). Collectively, these results suggest that the KRAS mutant regulates global mRNA m^6^A methylation by modulating ALKBH5 PTMs. Moreover, KRAS-mutant–driven platinum resistance in NSCLC correlates with KRAS-mutant–induced ALKBH5 PTMs.

### Blocking ALKBH5 SUMOylation overcomes platinum resistance of NSCLC cells.

**Figure 3:**
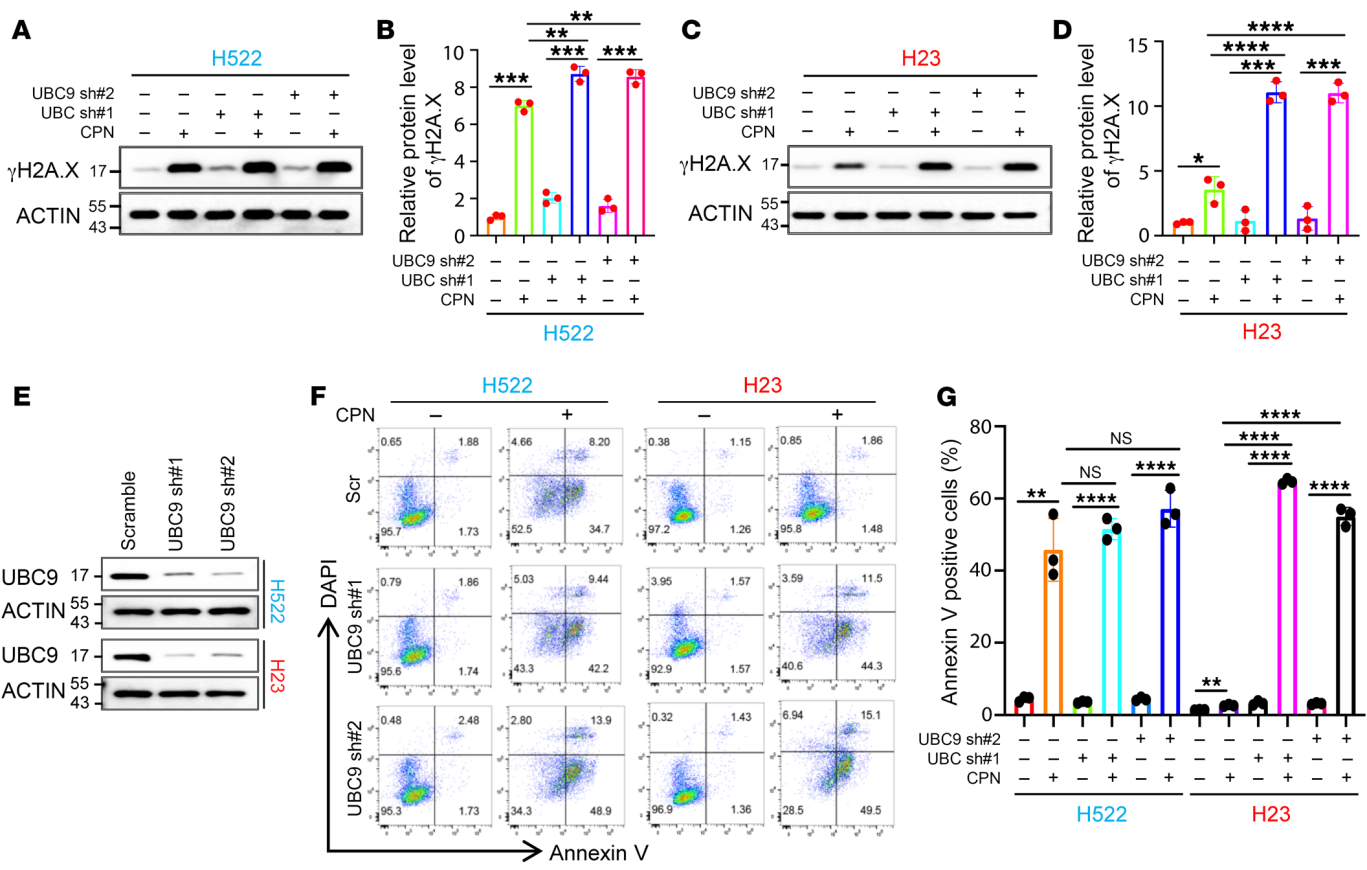
Blocking ALKBH5 SUMOylation overcomes platinum resistance of NSCLC cells. (**A**) Western blot analysis showing the effect of ALKBH5 SUMOylation blocking by UBC9 KD on the cisplatin sensitivity of *KRAS* WT NCI-H522 cells. (**B**) Histograms showing the summary and statistical analysis of the gray value of western bands shown in **A**. (**C**) Western blot analysis showing the effect of ALKBH5 SUMOylation blocking by UBC9 KD on the cisplatin sensitivity of *KRAS*-mutant NCI-H23 cells. (**D**) Histograms showing the summary and statistical analysis of the gray value of western bands shown in **C**. (**E**) Western blot analysis showing the KD efficiency of UBC9 in both NCI-H522 and NCI-H23 cells. (**F**) Cell apoptosis analysis suggests that blocking ALKBH5 SUMOylation by *UBC9* KD significantly sensitizes *KRAS*-mutant NCI-H23 cells to cisplatin-induced cell apoptosis. (**G**) Histograms showing the summary and statistical analysis of the data shown in **F**. In **B**, **D**, and **G**, data are presented as mean ± SD, with ordinary 1-way ANOVA with Dunnett’s multiple-comparison test used. **P < 0.01; ***P < 0.001; ****P < 0.0001.

Based on the aforementioned observations, we conducted a comparison of cisplatin-induced ALKBH5 PTMs between KRAS WT NCI-H522 and KRAS-mutant NCI-H23 cells. Notably, phosphorylation of ERK and JNK, as well as phosphorylation and SUMOylation of ALKBH5, were more significantly induced by cisplatin in NCI-H23 cells as compared with NCI-H522 cells (Figure 2L and Supplemental Figure 3J). In contrast, the levels of cisplatin-induced γH2A.X in NCI-H522 cells were considerably higher than those in NCI-H23 cells (Supplemental Figure 3J). These results indicate that the KRAS mutant promotes chemoresistance in lung cancer cells, a phenomenon correlated with the upregulation of ERK/JNK signaling as well as increased ALKBH5 phosphorylation and SUMOylation. To further confirm that the KRAS mutant confers drug resistance via ALKBH5 SUMOylation in NSCLC cells, we inhibited ALKBH5 SUMOylation in both NCI-H522 and NCI-H23 cells by knocking down SUMO E2 UBC9. The results showed that KRAS-mutant NCI-H23 cells are more sensitive to UBC9 depletion as compared with KRAS WT NCI-H522 cells and inhibition of ALKBH5 SUMOylation markedly enhances cisplatin-induced DNA damage and cell apoptosis in KRAS-mutant cells (Figure 3, A–G). Together, these findings strongly suggest that cisplatin-induced ALKBH5 PTMs play important roles in drug resistance conferred by KRAS mutants.

### Global transcriptomic and epitranscriptomic analyses identified NER-related genes including DDB2 and XPC as key downstream target genes of the KRAS mutant.

**Figure 4:**
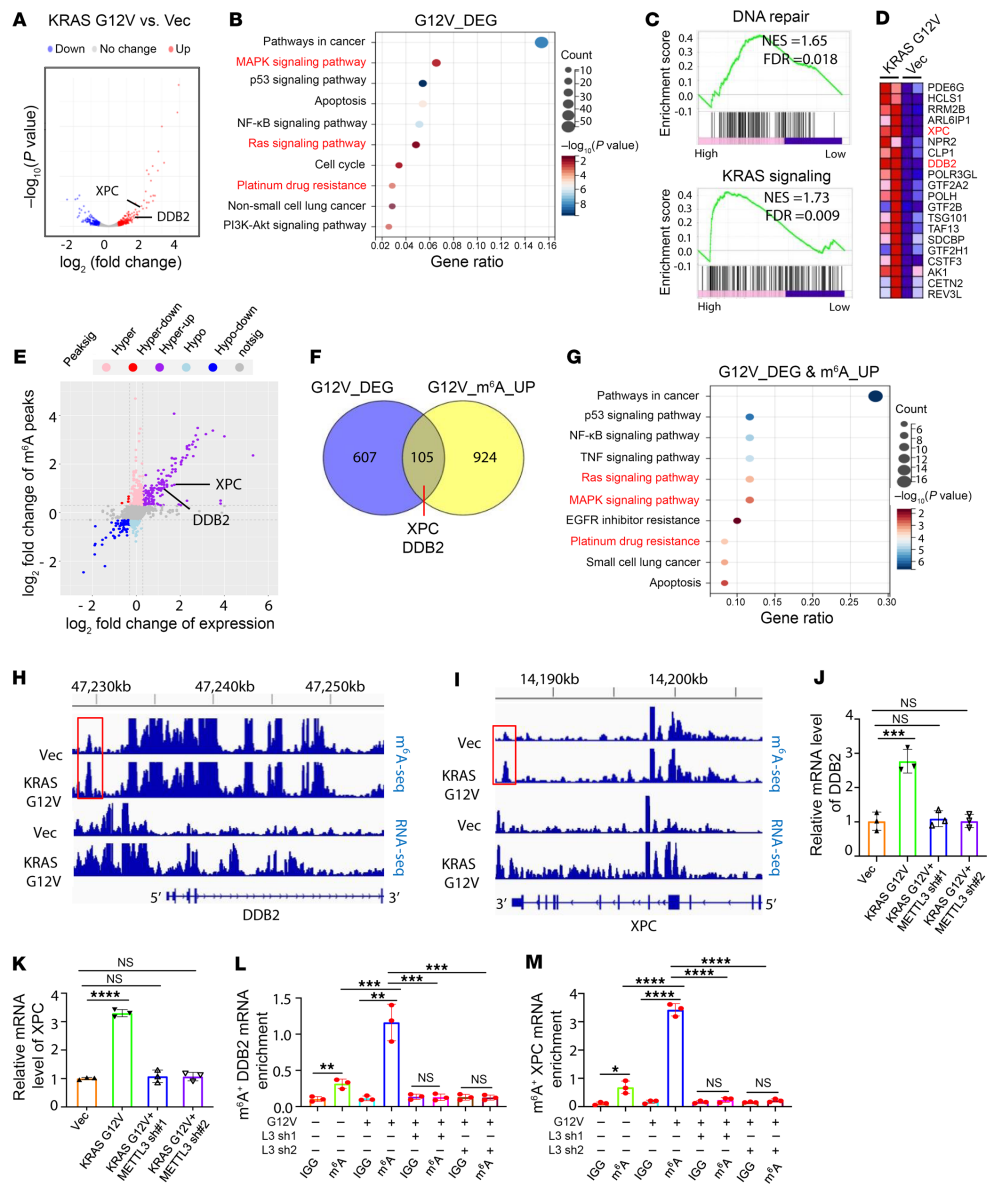
Global transcriptomic and epitranscriptomic analyses identified NER-related genes including *DDB2* and *XPC* are key downstream target genes of the *KRAS* mutant. (**A**) Volcano figure showing the differentially expressed genes induced by KRAS G12V overexpression in NCI-H522 cells. (**B**) GO analysis of the differentially expressed genes induced by KRAS G12V overexpression. (**C**) GSEA plot showing enrichment of gene sets of DNA damage repair and KRAS signaling in KRAS G12V–overexpressed NCI-H522 cells. (**D**) Heatmap showing the increased gene list of DNA damage repair–related genes induced by KRAS G12V overexpression shown in **C**. (**E**) Distribution of genes identified by m^6^A-seq with significant changes in both mRNA m^6^A methylation and overall expression induced by KRAS G12V overexpression. (**F**) Venn diagram shows the overlapped genes with both significant expression and m^6^A alterations upon KRAS G12V overexpression. (**G**) GO analysis of KRAS G12V downstream target genes in an m^6^A-dependent manner, identified by integrative analysis of RNA-Seq and m^6^A-Seq data in NCI-H522 cells. (**H** and **I**) RNA-Seq and m^6^A-Seq peak visualization of *DDB2* and *XPC* transcripts in empty vector– and KRAS G12V–overexpressed NCI-H522 cells. (**J** and **K**) qRT-PCR analysis suggests that KRAS G12V overexpression–mediated upregulation of *DDB2* and *XPC* could be rescued by METTL3 KD. (**L** and **M**) MeRIP analyses suggest that KRAS G12V overexpression–induced upregulation of m^6^A methylation levels of *DDB2* and *XPC* transcripts is blocked by METTL3 depletion. In **J**–**M**, data are presented as mean ± SD, with ordinary 1-way ANOVA with Dunnett’s multiple-comparison test used. **P < 0.01; ***P < 0.001; ****P < 0.0001.

To further explore the molecular mechanism underlying KRAS-mutant–mediated platinum resistance in lung cancer, we performed RNA-Seq analysis in control and KRAS G12V–expressing NCI-H522 cells. As shown in Figure 4A, KRAS G12V led to significant alterations in gene expression, with 429 and 283 genes upregulated and downregulated, respectively (log_2_FC > 0.3 or log_2_FC< –0.3, *P* < 0.05). GO analysis of those 712 differentially expressed genes induced by KRAS G12V revealed that the downstream target genes of the KRAS mutant are enriched in pathways involved in RAS and MAPK signaling pathways and platinum resistance, as well as pathways in cancer (Figure 4B). Additionally, gene set enrichment analysis (GSEA) analyses revealed that the downstream target genes of the KRAS mutant are enriched in pathways involved in RAS signaling and DNA damage repair (Figure 4, C and D). By integrative analysis of RNA-Seq and m^6^A-Seq data, 105 genes were differentially expressed with an upregulation of m^6^A methylation level upon KRAS G12V expression. GO analysis of these genes revealed that those genes are also enriched in the pathways involved in the activation of RAS and MAPK signaling as well as platinum resistance (Figure 4, F and G). Among these genes, DDB2 and XPC stood out due to their important roles in multiple pathways that regulate the NER and platinum resistance (22, 23) (Figure 4, D, F, and G). Notably, both the m^6^A methylation and expressions of DDB2 and XPC are significantly induced by KRAS G12V (Figure 4, A, E, H, and I). Consistent with the RNA-Seq and m^6^A-Seq results, both the transcription and mRNA m^6^A methylation levels of DDB2 and XPC were markedly increased by KRAS G12V, as determined by quantitative reverse transcriptase PCR (qRT-PCR) and the methylated RNA IP (MeRIP) followed by RT-PCR analyses, respectively. METTL3 KD, which blocks KRAS G12V–induced m^6^A methylation, significantly inhibited DDB2 and XPC expression (Figure 4, J–M), suggesting that the KRAS mutant regulates DDB2 and XPC mRNA expression in an m^6^A-dependent manner. Notably, KRAS G12V–induced upregulation of DDB2 and XPC was reversed by overexpression of a SUMOylation-deficient mutant ALKBH5 but not WT ALKBH5, suggesting that KRAS mutant regulates DDB2 and XPC expression through ALKBH5 SUMOylation (Supplemental Figure 4, A and B). We next investigated whether the KRAS-mutant drove platinum resistance, at least partially through the induction of DDB2 and XPC expression. As illustrated in Supplemental Figure 4, C and D, cisplatin treatment significantly induced the expression of DDB2 and XPC; and KRAS G12V further augmented cisplatin-induced expression of these genes in BEAS-2B cells (Supplemental Figure 4, C and D). In addition, cisplatin significantly induced expression of DDB2 and XPC in KRAS WT NCI-H522 cells (Supplemental Figure 4, E and F). Notably, the induction of expression of these genes was more significant in KRAS-mutant NCI-H23 cells compared with NCI-H522 cells (Supplemental Figure 4, E and F). These results suggest that the enhanced NER pathway with upregulation of DDB2 and XPC likely contributes to the resistance to chemotherapeutic drug in KRAS G12C-mutant NCI-H23. Collectively, these results indicate that the KRAS mutant induces chemoresistance possibly by facilitating the expression of NER-related genes, including DDB2 and XPC, in an m^6^A-dependent manner in NSCLC cells.

### Cisplatin/KRAS-induced m^6^A modification of DDB2 and XPC lead to their mRNA stabilization.

**Figure 5:**
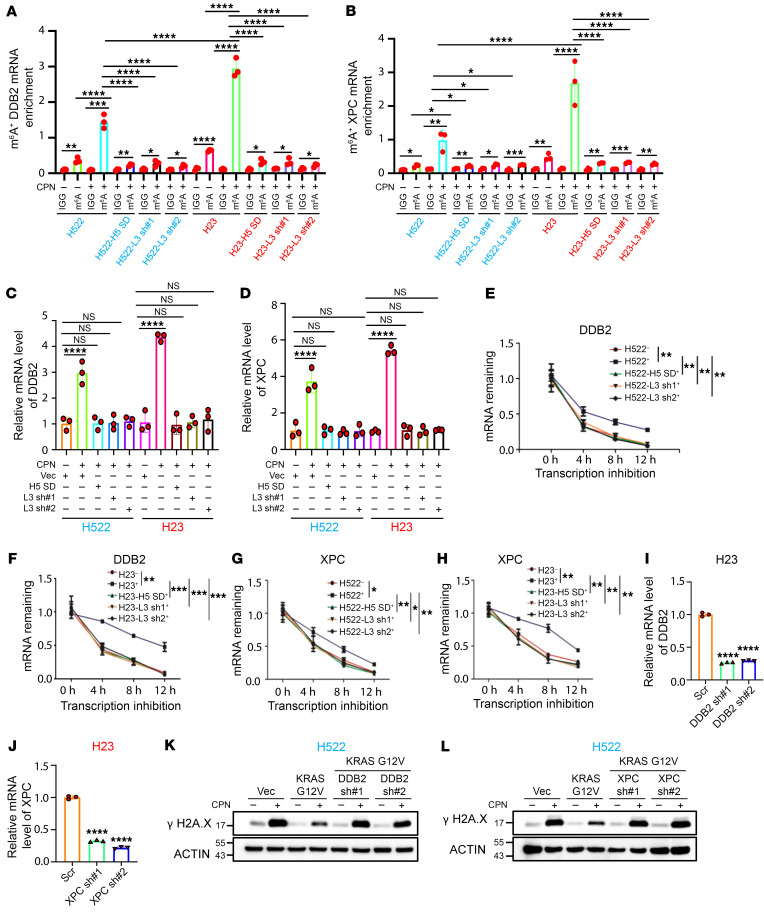
Cisplatin/KRAS-induced m^6^A modification of *DDB2* and *XPC* lead to their mRNA stabilization. (**A** and **B**) MeRIP analyses showing mRNA m^6^A levels of *DDB2* and *XPC* in the NSCLC cells, as indicated. (**C** and **D**) qRT-PCR analysis for *DDB2* and *XPC* in the cell lines as indicated. (**E**–**H**) Analysis of mRNA half-lives of *DDB2* and *XPC* in the NSCLC cells as indicated. (**I** and **J**) qRT-PCR analysis showing the KD efficiency of *DDB2* and *XPC* in NCI-H23 cells, respectively. (**K** and **L**) Western blot analyses suggest that either DDB2 or XPC KD significantly sensitizes KRAS G12V–overexpressed NCI-H522 cells to cisplatin treatment. In **A**–**J**, data are presented as mean ± SD, with ordinary 1-way ANOVA with Dunnett’s multiple-comparison test used. *P < 0.05; **P < 0.01; ***P < 0.001; ****P < 0.0001.

We next investigated the interplay between cisplatin-induced gene expression and the elevated m^6^A methylation levels of *DDB2* and *XPC*. As depicted in Figure 5, A and B, either expression of a SUMOylation-deficient mutant ALKBH5 or METTL3 KD by 2 specific shRNAs effectively blocked the cisplatin-induced m^6^A methylation increase of *DDB2* and *XPC*, leading to a downregulation of both genes in both NCI-H522 and NCI-H23 cells (Figure 5, C and D). Increased m^6^A methylation levels of *DDB2* and *XPC* in *KRAS*-mutant NCI-H23 resulted in the prolonged half-lives of *DDB2* and *XPC* mRNA compared with *KRAS* WT NCI-H522 cells. Cisplatin treatment markedly enhanced the stability of *DDB2* and *XPC* mRNA in *KRAS*-mutant NCI-H23 cells compared with *KRAS* WT NCI-H522 cells. Notably, the prolonged half-lives of *DDB2* and *XPC* mRNA induced by cisplatin in NCI-H522 and NCI-H23 cells were entirely reversed by expression of the SUMOylation-deficient ALKBH5 or by METTL3 KD (Figure 5, E–H). Similarly, either pharmacological inhibition of KRAS G12C or ERK completely reversed the prolonged mRNA half-lives of *DDB2* and *XPC* in KRAS G12C harboring H23 cells (Supplemental Figure 4, G–J). Thus, these results suggest that cisplatin-induced m^6^A methylation of *DDB2* and *XPC* leads to stabilization of their mRNA, which can be further augmented by the KRAS mutant in NSCLC cells.

### KRAS mutations confer platinum resistance in NSCLC cells by modulating DDB2- and XPC-mediated NER.

Next, we aimed to uncover the mechanism underlying KRAS/ERK/ALKBH5 PTMs/ DDB2 and XPC signaling axis-mediated platinum resistance in NSCLC cells. Given that both DDB2 and XPC are key components of NER machinery, we sought to determine whether the NER pathway is involved in KRAS mutation–driven platinum resistance in lung cancer. Consistent with previous studies (23, 43), KD of either *DDB2* or *XPC* significantly reduced NER activity in NCI-H23 cells (Supplemental Figure 5, A–D). Notably, NER activity was significantly higher in *KRAS*-mutant NSCLC cells compared with *KRAS* WT lung cancer cells (Supplemental Figure 5, E and F), suggesting a positive correlation between *KRAS* mutations and NER activity in NSCLC cells. Additionally, KRAS G12V overexpression significantly enhanced NER activity in *KRAS* WT H522 cells (Supplemental Figure 5, G and H). Conversely, NER activity in *KRAS*-mutant H23 cells was significantly inhibited by KRAS KD (Supplemental Figure 5, I and J). Together, these data provide compelling evidence that *KRAS* mutations positively regulate NER activity in NSCLC cells. Moreover, as shown in Supplemental Figure 6, A–F, KD of either *DDB2* or *XPC* significantly sensitized *KRAS*-mutant H23 cells to cisplatin-induced DNA damage. Furthermore, KRAS G12V overexpression–induced H522 cisplatin resistance was completely blocked by KD of either DDB2 or XPC (Figure 5, I–L). Collectively, these results suggest that *DDB2* and *XPC* play key roles in *KRAS* mutation-driven platinum resistance in NSCLC cells and that KRAS mutations confer drug resistance by enhancing NER activity.

### ALKBH5 SUMOylation serves as a direct functional mediator in KRAS mutation-driven platinum resistance in NSCLC cells.

RNA m^6^A methylation is dynamically regulated by m^6^A writer, of which the major catalytic subunit is METTL3, and erasers, including ALKBH5 and FTO (13, 35). Therefore, we investigated whether KRAS mutation–driven platinum resistance involves the regulation of FTO or METTL3 expression. Interestingly, KRAS G12V overexpression did not affect the protein levels of FTO or its PTMs, including phosphorylation and SUMOylation (Supplemental Figure 7A). Similarly, cisplatin resistance of *KRAS*-mutant H23 cells could not be overcome by FTO overexpression (Supplemental Figure 7B). Consistently, neither the cisplatin-induced expression nor the m^6^A methylation of *DDB2* and *XPC* was restored by FTO overexpression (Supplemental Figure 7, C–F), suggesting that DDB2 and XPC, as functional mediators of KRAS mutations, are specific downstream targets of ALKBH5. Moreover, KRAS G12V overexpression significantly upregulated METTL3 expression (Supplemental Figure 7, G and H). However, both KRAS G12V– and cisplatin-induced METTL3 expression were completely reversed by overexpression of a SUMOylation-deficient mutant ALKBH5 (Supplemental Figure 7, H and I), indicating that KRAS mutations induce METTL3 expression by regulating ALKBH5 SUMOylation. Collectively, these findings suggest that KRAS-mutant–driven platinum resistance in NSCLC cells is mediated directly through the regulations of ALKBH5 SUMOylation. Furthermore, DDB2 and XPC, identified as functional mediators of KRAS mutants, are specific downstream targets of ALKBH5.

### The KRAS mutant confers NSCLC drug resistance by hijacking AKBH5 PTM-mediated DNA repair pathways in vivo.

**Figure 6:**
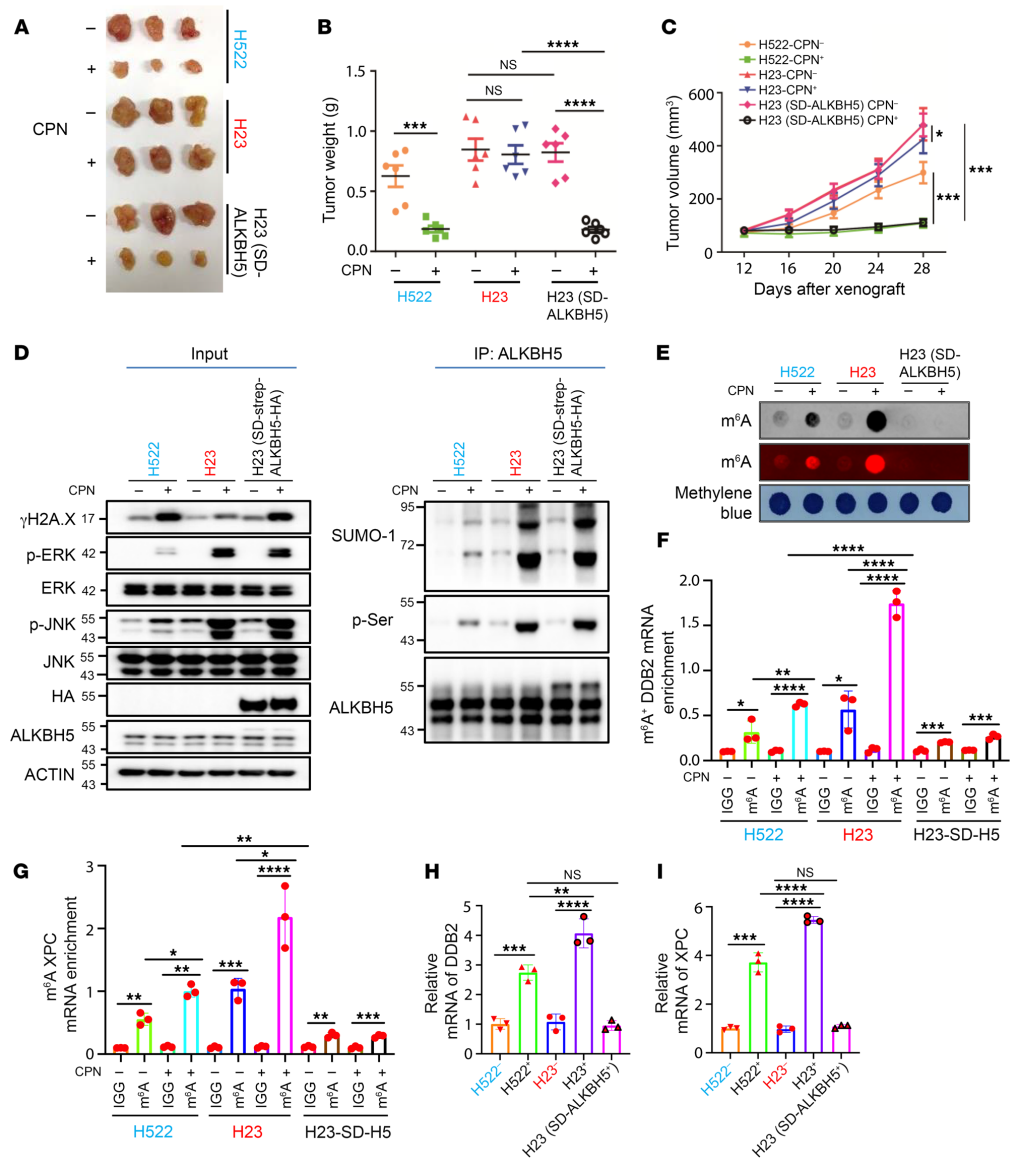
KRAS constitutively active mutation confers NSCLC drug resistance by hijacking AKBH5 PTM–mediated DNA repair pathways in vivo. (**A**–**C**) Effects of cisplatin injection and overexpression of SUMOylation-deficient mutant ALKBH5 (SD-ALKBH5) on tumor growth of NCI-H522 and NCI-H23 xenograft mice. *n* = 3 mice for each group, and lung cancer cells as indicated were injected at 2 flanks of each mouse. (**D**) Denature IP analysis showing the ALKBH5 PTM levels in the indicated lung cancer xenografts. Proteins were extracted from 3 tumors, each obtained from a different mouse, and then combined into a single mixture for the IP analysis. (**E**) Dot-blot analysis suggesting global mRNA m^6^A levels in the xenografts as indicated. RNAs were extracted from 3 tumors, each obtained from a different mouse, and then combined into a single mixture for the dot-blot analysis. (**F** and **G**) MeRIP analysis showing mRNA m^6^A levels of *DDB2* and *XPC* in the xenografts as indicated. (**H** and **I**) qRT-PCR analysis indicating transcription levels of *DDB2* and *XPC* in the xenografts as indicated. In **B**, **C**, and **F**–**I**, data are presented as mean ± SD, with ordinary 1-way ANOVA with Dunnett’s multiple-comparison test used. *P < 0.05, **P < 0.01, ***P < 0.001, ****P < 0.0001.

To further determine whether KRAS mutation confers NSCLC drug resistance through the KRAS/ERK/JNK/ALKBH5 PTMs/m^6^A/DDB2 and XPC/NER signaling axis in vivo, we carried out xenograft experiments with NSCLC cells. As shown in Figure 6, A–C, KRAS-mutant NCI-H23 cells were more resistant to cisplatin treatment compared with KRAS WT NCI-H522 in vivo. Notably, ectopic expression of SUMOylation-deficient mutant ALKBH5 (SD-ALKBH5) substantially sensitized NCI-H23 cells to cisplatin treatment in vivo. Consistent with previously published studies (44, 45), the toxic effect of cisplatin treatment was minimal in our experimental settings, as evidenced by the stable mouse weights and unaltered xenograft growth (Supplemental Figure 7J). In addition, ERK/JNK signaling was significantly more activated, resulting in lower levels of DNA damage in KRAS-mutant NCI-H23 cells in the xenograft model with cisplatin treatment compared with *KRAS* WT NCI-H522 xenografts (Figure 6D). Expression of SUMOylation-deficient mutant ALKBH5 (SD-ALKBH5) substantially facilitated cisplatin-induced DNA damage in NCI-H23 xenografts (Figure 6D). Consistently, ALKBH5 PTMs, including phosphorylation and SUMOylation, are significantly more pronounced in response to cisplatin treatment in *KRAS*-mutant H23 cells compared with *KRAS* WT H522 cells in vivo (Figure 6D). Moreover, global mRNA m^6^A methylation levels were induced more significantly in NCI-H23 xenografts by cisplatin treatment as compared with NCI-H522 xenografts (Figure 6E). SUMOylation-deficient mutant ALKBH5 (SD-ALKBH5) overexpression completely blocked cisplatin-induced mRNA m^6^A methylation in NCI-H23 xenografts (Figure 6E). More importantly, cisplatin treatment significantly induced m^6^A methylation of *DDB2* and *XPC* in *KRAS* WT NCI-H522 xenografts (Figure 6, F and G). The induction of m^6^A methylation levels of these genes was even more pronounced in *KRAS*-mutant NCI-H23 xenografts (Figure 6, F and G). Importantly, the cisplatin-induced m^6^A methylation of *DDB2* and *XPC* genes in *KRAS*-mutant NCI-H23 xenografts was blocked by overexpression of the SUMOylation-deficient mutant ALKBH5 (SD-ALKBH5) (Figure 6, F and G). Consistently, the expression levels of *DDB2* and *XPC* were higher in NCI-H23 xenografts than in NCI-H522 xenografts with cisplatin treatment (Figure 6, H and I). Overexpression of SUMOylation-deficient mutant ALKBH5 (SD-ALKBH5) blocked cisplatin-induced upregulation of *DDB2* and *XPC* in NCI-H23 xenografts (Figure 6, H and I). Collectively, these results indicate that the KRAS mutant promotes platinum resistance in NSCLC cells in vivo by hijacking ALKBH5 PTM–mediated DNA repair pathways.

### METTL3 inhibition sensitizes KRAS-mutant NSCLC cells to cisplatin in vivo.

**Figure 7:**
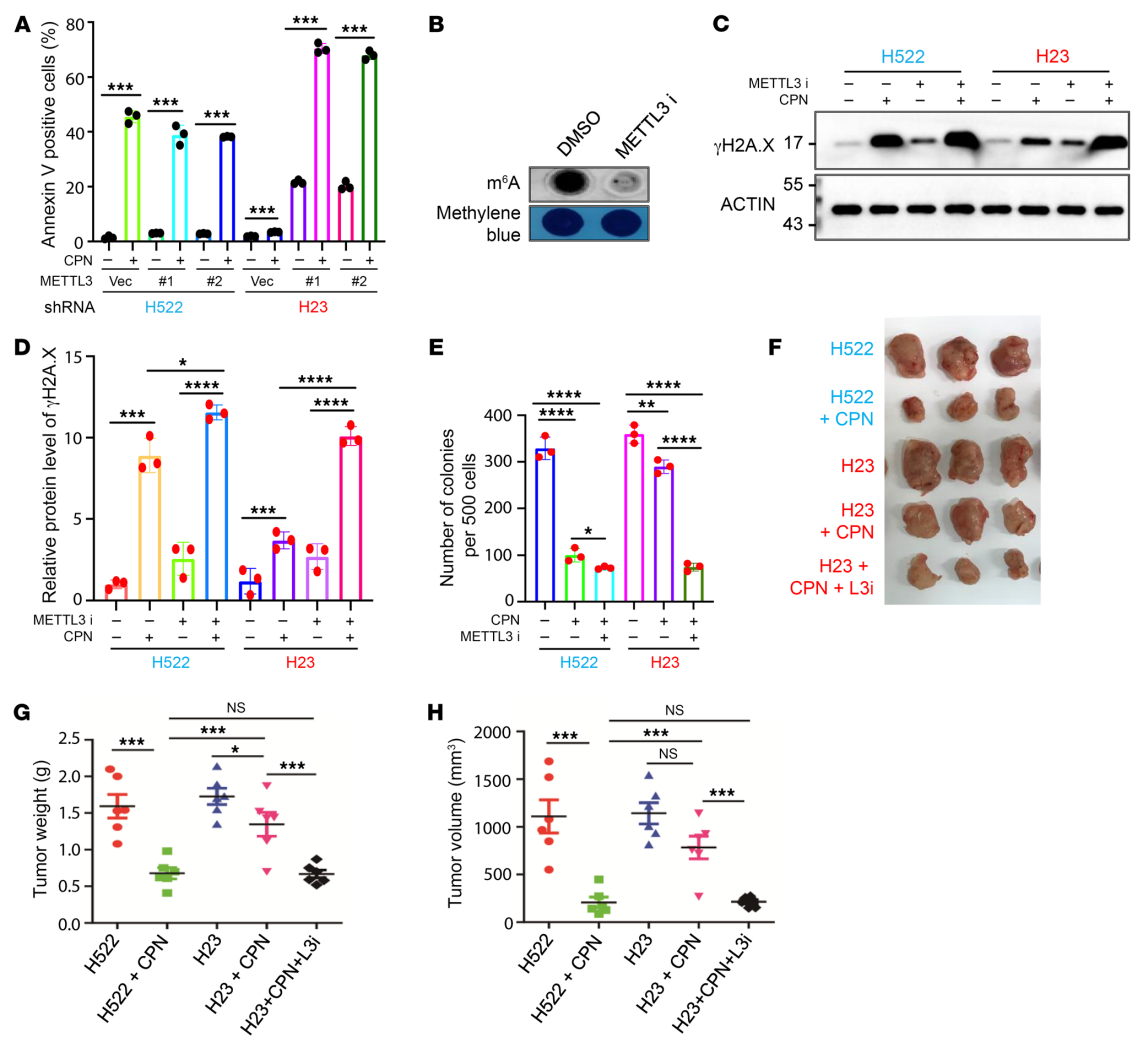
METTL3 inhibition sensitizes *KRAS* mutation harboring NSCLC cells to cisplatin in vivo. (**A**) Annexin V staining analysis for the NSCLC cells as indicated. (**B**) Dot-blot analysis showing the effect of METLL3 inhibition on global mRNA m^6^A methylation levels in NCI-H23 cells. (**C**) Western blot analysis indicates that METTL3 inhibition by 10 μM STM2457 significantly sensitizes *KRAS* mutation harboring NCI-H23 cells to cisplatin-induced DNA damage. (**D**) Histograms showing the summary and statistical analysis of the gray value of western bands shown in **C**. (**E**) Colony-forming analysis for the NSCLC cells as indicated. (**F**–**H**) NSCLC xenograft experiments suggest that pharmacological inhibition of METTL3 markedly sensitizes *KRAS*-mutant NCI-H23 cells to cisplatin treatment in vivo. *n* = 3 mice for each group, and lung cancer cells as indicated were injected at 2 flanks of each mouse. In **A**, **D**, **E**, **G**, and **H**, data are presented as mean ± SD, with ordinary 1-way ANOVA with Dunnett’s multiple-comparison test used for **D**, **E**, **G** and **H** and 2-tailed Student’s *t* test for **A**. *P < 0.05, **P < 0.01, ***P < 0.001, ****P < 0.0001.

To investigate whether *METTL3* KD exerts a similar rescue phenotype as ectopic expression of SD-ALKBH5, we established stable lines of NCI-H522 and NCI-H23 cells expressing scramble control or *METTL3*-specific shRNAs. As shown in Supplemental Figure 8 A and B, METTL3 was significantly KD by both specific shRNAs. *METTL3* KD exhibited greater sensitivity to cisplatin-induced DNA damage and cell apoptosis in *KRAS*-mutant NCI-H23 cells compared with *KRAS* WT NCI-H522 cells (Figure 7A and Supplemental Figure 8, C–E). To assess the potential therapeutic application of targeting METTL3 in *KRAS*-mutant NSCLC cells, we employed a small molecule, STM2457, which potently and selectively inhibited METTL3 enzymatic activity in a recent study (46). Consistently, METTL3 inhibition by STM2457 markedly inhibited global mRNA m^6^A methylation in *KRAS*-mutant NCI-H23 cells (Figure 7B). Similar to METTL3 KD, METTL3 inhibition significantly sensitized NCI-H23 cells to cisplatin-induced DNA damage (Figure 7, C and D). Notably, γ-H2AX levels were increased upon METTL3 inhibition in NSCLC cells. METTL3 inhibition reduced m^6^A methylation in NER-related genes, such as DDB2 and XPC, resulting in their mRNA decay and subsequent suppression of NER activity. Additionally, METTL3 inhibition significantly enhanced the cisplatin-mediated suppression of the colony-forming ability of KRAS-mutant NCI-H23 cells (Figure 7E and Supplemental Figure 8F), and it markedly increased the sensitivity of NCI-H23 cells to cisplatin treatment in vivo (Figure 7, F–H). Meanwhile, in vivo METTL3 inhibition using STM2457 demonstrated minimal toxicity. Over a 40-day monitoring period, STM2457 injection didn’t cause acute mortality or substantial body weight loss in mice, nor did it visibly affect the morphology of major organs. Collectively, these results suggest that METTL3 is a promising and safe target for sensitizing *KRAS*-mutant NSCLC to cisplatin treatment.

### KRAS mutants confer NSCLC drug resistance in primary lung cancer cells from patients.

**Figure 8:**
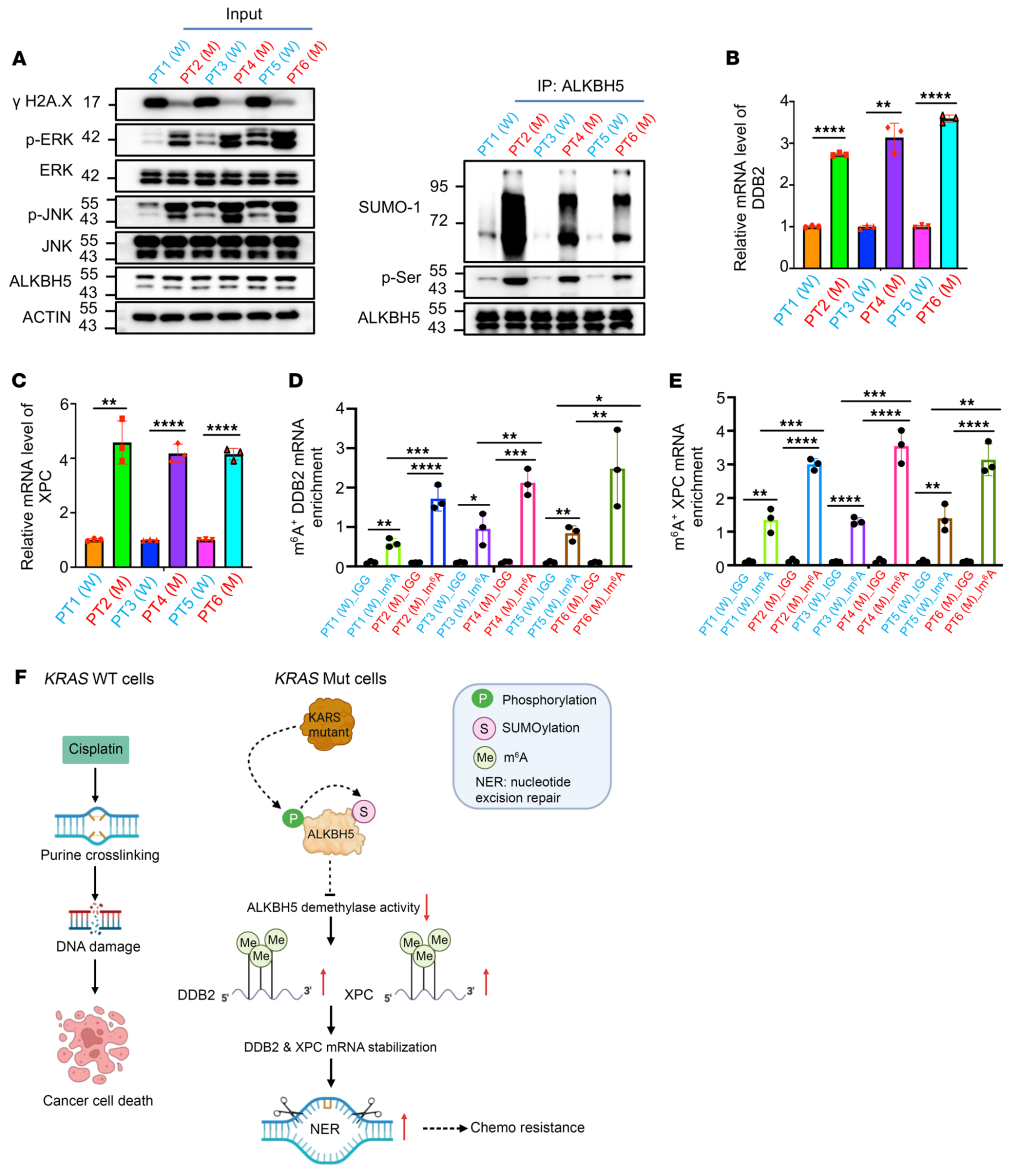
KRAS/ERK/JNK/ALKBH5 PTMs/m^6^A/DDB2 and XPC/NER signaling axis occurs frequently among clinical lung cancer patients. (**A**) Western blot analysis showing the protein levels as indicated in the indicated clinical platinum-based chemotherapeutic lung cancer samples. (**B** and **C**) qRT-PCR analysis showing the mRNA levels of *DDB2* and *XPC* in *KRAS* WT and mutant lung cancer patient samples. (**D** and **E**) MeRIP analysis showing the mRNA m^6^A levels of *DDB2* and *XPC* in *KRAS* WT and mutant lung cancer patient samples. (**F**) Working model of KRAS-mutant–mediated platinum resistance in NSCLC. In *KRAS* WT lung cancer cells, cisplatin treatment causes DNA damage by inducing purine nucleotide crosslinking, ultimately triggering apoptosis. However, in *KRAS*-mutant lung cancer cells, KRAS mutations activate ERK/JNK signaling, leading to ALKBH5 phosphorylation and subsequent SUMOylation. This SUMOylation inhibits its m^6^A demethylase activity, leading to a global increase in mRNA m^6^A methylation, including on NER-related genes such as *DDB2* and *XPC*. The stabilization of *DDB2* and *XPC* mRNA enhances NER, allowing KRAS mutations to drive chemoresistance. In **B**–**E**, data are presented as mean ± SD, with ordinary 1-way ANOVA with Dunnett’s multiple-comparison test used for **D** and **E** and 2-tailed Student’s *t* test for **B** and **C**. *P < 0.05; **P < 0.01; ***P < 0.001; ****P < 0.0001.

To further determine whether the aforementioned observations also exist in the primary lung cancer cells from patients, we collected 3 pairs of platinum-based chemotherapeutic primary lung adenocarcinoma tissues, both *KRAS* WT and mutant, from patients at University of Florida Shands Hospital. As shown in Figure 8A, the ERK/JNK signaling is more significantly activated, resulting in lower levels of DNA damage in *KRAS*-mutant lung cancer cells compared with *KRAS* WT lung cancer cells from patients. Consistently, ALKBH5 PTMs, including phosphorylation and SUMOylation, are much more abundant in *KRAS*-mutant primary lung cancer cells compared with *KRAS* WT cells (Figure 8A). Moreover, qRT-PCR analyses showed that both the *DDB2* and *XPC* genes were expressed at much higher levels in primary *KRAS*-mutant lung cancer cells compared with primary *KRAS* WT lung cancer cells (Figure 8, B and C). In addition, the m^6^A methylation levels of *DDB2* and *XPC* transcripts were also higher in primary *KRAS*-mutant lung cancer cells compared with primary *KRAS* WT lung cancer cells (Figure 8, D and E). These findings suggest that the identified KRAS/ERK/JNK/ALKBH5 PTMs/m^6^A/DDB2 and XPC/NER signaling axis are also active in primary lung cancer cells from patients. KRAS mutants confer platinum resistance at least partially through the posttranscriptional regulation of *DDB2* and *XPC* in an m^6^A-dependent manner, thereby facilitating the nucleotide excision of the crosslinked purine nucleotides induced by platinum-based chemotherapy drugs.

## Discussion

Despite numerous therapeutic strategies having been developed for clinical lung cancer patient treatment, including surgical treatment, immunotherapy, radiation, and chemotherapy, chemotherapy is still the critical component of the treatment regimen for the patients with NSCLC (6, 47–49). The efficacy of chemotherapy in *KRAS* mutant NSCLC patients is poor (50). The significance of *KRAS* as a prognosis marker in NSCLC is controversial (50). It was reported that *KRAS*-mutant NSCLC patients responded more poorly to cytotoxic therapy compared with *EGFR* WT/*KRAS* WT patients (9, 10). Platinum-based drugs exert their therapeutic effects by crosslinking purine bases on DNA, disrupting DNA repair processes, causing DNA damage, and subsequently triggering cell apoptosis. Our studies demonstrated that *KRAS*-mutant NSCLC cells are more resistant to cisplatin treatment in vitro and in vivo. More importantly, we provide compelling evidence supporting that KRAS mutants confer NSCLC platinum resistance via inducing upregulation of m^6^A methylation of DNA repair genes, particularly *DDB2* and *XPC*. An increase of m^6^A methylation in *DDB2* and *XPC* transcripts leads to upregulation of DDB2 and XPC expression through stabilizing their mRNAs. Consequently, the increased DDB2 and XPC expression led to the accelerated excision of the crosslinked purine nucleotides, thereby conferring NSCLC platinum resistance. Upon cisplatin treatment, KD of either the *DDB2* or *XPC* gene increased DNA damage and induced apoptosis in *KRAS*-mutant NSCLC cells, thereby sensitizing these cells to cisplatin treatment. In addition, we showed that *KRAS*-mutants or *KRAS* KD do not affect the expression of ABC transporters including ABCB1, ABCG2, and ABCC1 in NSCLC cells, ruling out the possibility that KRAS-mutant–mediated NSCLC platinum resistance is a result of the dysregulation of ABC transporters. Moreover, we found that *KRAS*-mutant NSCLC cells are not resistant to PTX, which is also a frequently used chemotherapeutic drug in lung cancer treatment (28–31). Thus, our data suggest that *KRAS*-mutant NSCLC cells are specifically resistant to cisplatin but not to PTX, compared with *KRAS*-WT NSCLC cells. Additionally, the DDB2- and XPC-mediated NER pathway likely plays an important role in platinum-based chemoresistance. Notably, the KRAS mutant induces differential expression of over a hundred genes through upregulating m^6^A methylation of these genes, which are involved in the RAS and MAPK signaling pathway and platinum resistance. Thus, additional molecular pathways may also contribute to KRAS-mutant–mediated chemoresistance.

In this study, we uncovered a role of KRAS in regulating mRNA m^6^A methylation through regulating ALKBH5 PTMs in NSCLC cells. Although a previous study suggests that RAS/MAPK signaling regulates global mRNA m^6^A methylation through EKR-mediated phosphorylation of METTL3, thereby facilitating METTL3 protein stabilization by increasing USP5-mediated deubiquitination (51), our current study suggests that *KRAS*-mutants also regulate mRNA m^6^A methylation through inactivating ALKBH5 m^6^A demethylase activity by inducing ALKBH5 phosphorylation and SUMOylation.

Cisplatin treatment has been shown to induce oxidative stress, activating a DNA damage response through ROS (52–57). Our previous study (35) demonstrated that ROS activates ERK/JNK signaling, leading to the phosphorylation of ALKBH5 at serine 325. This phosphorylation recruits the SUMO E2 enzyme UBC9, promoting ALKBH5 SUMOylation at lysine residues K86 and K321, which inhibits its m^6^A demethylase activity and upregulates genes involved in DNA damage repair.

In this study, we show that constitutively active *KRAS* mutations also induce ALKBH5 phosphorylation at serine 325, triggering its SUMOylation at the same lysine residues. This inactivates ALKBH5 and upregulates NER-related genes, such as *DDB2* and *XPC*, in an m^6^A-dependent manner, enhancing cisplatin resistance in NSCLC cells. Both ROS and KRAS mutations increase DNA repair capabilities by regulating ALKBH5 PTMs. Given that several studies (58, 59) suggest that KRAS overexpression also induces ROS production, KRAS-mediated ROS generation may also contribute to the KRAS mutation–driven platinum resistance in NSCLC cells.

Notably, the ALKBH5 PTM sites induced by ROS and KRAS mutations are identical, suggesting a synergistic effect between ROS and KRAS mutations in driving platinum resistance, further reducing NSCLC cell sensitivity to cisplatin. Our findings are supported by evidence: (a) KRAS G12C–induced ALKBH5 phosphorylation and SUMOylation were blocked by KRAS G12C or ERK inhibitors, confirming that RAS/ERK signaling is essential for KRAS-driven ALKBH5 PTMs; (b) KRAS G12V overexpression induced SUMOylation of WT ALKBH5 but not the phosphorylation-deficient mutant S325D, indicating that SUMOylation depends on phosphorylation; and (c) ROS-triggered ALKBH5 phosphorylation, as shown in our previous study (35), leads to its SUMOylation via ERK/JNK signaling, and KRAS-induced PTMs occur at the same sites. However, the precise mechanism by which KRAS mutations induce ALKBH5 phosphorylation requires further investigation.

In conclusion, the interplay between oncogenic KRAS and ROS-mediated DNA damage response plays a critical role in the reduced sensitivity of KRAS-mutant NSCLC cells to platinum-based therapies. This underscores the importance of targeting the ERK/JNK/ALKBH5 PTM/NER signaling axis to overcome platinum resistance in these cells.

Despite being the most frequently mutated and activated oncogene in various cancers, targeting KRAS has posed a great therapeutic challenge over the past 50 years since its discovery. The development of small-molecule inhibitors relies on the availability of suitable binding pockets on the protein’s surface. KRAS, however, has long been considered “undruggable” due to the absence of such binding pockets (5, 60). Therefore, although KRAS was identified as an oncogene as early as 1969, only 2 drugs specifically targeting KRAS G12C have received FDA approval (6). Despite this success, there remains a big challenge of combating the resistance that NSCLC cells, xenografts, and patients have exhibited while being treated with KRAS G12C inhibitors (6). Furthermore, published studies have revealed various KRAS mutations, including KRAS G12C, G12A, G12D, G12V, G12S, G12R, G12F, G13C, G13D, and Q61R in NSCLC cells (7, 61). Unfortunately, the current developed inhibitors can only target KRAS G12C. Additionally, many attempts have been made to target KRAS downstream pathways, specifically, the MAPK and PI3K/AKT pathways (62, 63). For example, the small molecules developed, such as selumetinib, which directly targets MEK, showed early promise; however, further studies showed no statistically significant effects in *KRAS* mutant patients (63, 64). Therefore, treatment of *KRAS*-mutant lung cancer remains a challenge. Our current study suggests an alternative approach for the treatment. We found that blocking the cisplatin/*KRAS* mutation-induced m^6^A methylation through METTL3 inhibitor substantially enhances the sensitivity of *KRAS*-mutant NSCLC cells to cisplatin treatment, both in vitro and in vivo. This strategy allows us to combine METTL3 inhibitors with platinum-based drugs to treat the NSCLC cells, opening new avenues for the treatment of NSCLC patients.

In summary, our study has unraveled the intricate mechanisms through which KRAS mutations orchestrate the ERK/JNK signaling pathways, posttranslational modifications of ALKBH5, and mRNA m^6^A modification to confer platinum resistance in NSCLC cells. We have shed light on molecular mechanisms by which KRAS constitutively active mutations elevate mRNA m^6^A methylation, thus adding as a new layer of regulating ALKBH5 m^6^A demethylase activity, as well as gene regulation that fortifies DNA repair–related genes, shielding NSCLC cells from cisplatin-induced DNA damage and cell apoptosis. This ultimately facilitates chemoresistance in NSCLC (Figure 8F). Moreover, our research uncovered a mechanism by which KRAS mutants foster resistance to chemotherapy in NSCLC cells by hijacking ALKBH5 PTM–mediated DNA damage response pathways (Figure 8F). Finally, we found that combining cisplatin with a METTL3 inhibitor markedly sensitizes *KRAS* mutant NSCLC cells to cisplatin exposure, offering a promising strategy for the treatment of NSCLC.

## Methods

### Sex as a biological variable.

In all NSCLC triple transgenic NSG-SGM3 (NSGS) mouse xenograft studies, both male and female mice were used. Sex was not considered as a biological variable in the statistical analyses. The NSGS mice used for NSCLC xenograft studies were purchased from The Jackson laboratory.

### Cell lines.

Both the normal epithelial cells BEAS-2B, WT KRAS harboring NCI-H522 and NCI-H2087, and KRAS mutant NSCLC cells including NCI-H23, NCI-H2122, NCI-H1573, and NCI-H2009 were provided in house. The BEAS-2B cells were cultured in the BEGM Bronchial Epithelial Cell Growth Medium BulletKit (Lonza, catalog CC-3170). For routine maintenance, all the NSCLC cells were cultured at 37°C with 5% CO_2_ in RPMI-1640 containing 10% FBS and 1% penicillin/streptomycin.

### Plasmids and antibodies.

pCDH-Flag-KRAS G12V and pCDH-Flag-KRAS S17N were subcloned from plasmids provided in house. The pCDH-Strep-ALKBH5-HA expression plasmid was generated by cloning the corresponding coding sequence into the pCDH-Strep vector. All the pCDH-Strep-HA-ALKBH5 K/R (lysine to arginine) or S/A (serine to alanine) mutants were derived from pCDH-Strep-HA-ALKBH5 by site-directed mutagenesis. Information about antibodies used in this study is provided in Supplemental Table 1.

### Drug treatment.

For the lung cancer cell drug resistance analysis, the cells were treated with DMSO or 20 μM cisplatin for 24 hours. For the rescue experiment by METTL3 inhibition, the indicated cells were treated with 10 μM STM2457 for 24 hours. For KRAS G12C inhibition, NCI-H23 cells were treated with 0.1 μM sotorasib for 3 hours. For ERK inhibition, NCI-H23 cells were treated with 1 μM PD0325901 for 3 hours.

### Western blot analysis and co-IP.

The Western blot and co-IP analyses were performed according to standard protocols as described previously with minor changes (65), by using the antibodies as indicated. For examining SUMO-modified proteins, cells were lysed in denaturing buffer (50 mM Tris-HCl pH7.5, 150 mM NaCl, 4% SDS, 1mM EDTA, 8% glycerol, 50mM NaF, 1 mM DTT, 1mM phenylmethylsulfonyl fluoride (PMSF), and protease inhibitors) supplemented with 20 mM N-ethylmaleimide (NEM) and heated at 90°C for 10 minutes. For the following IP assays, the lysates were further diluted to 0.1% SDS and immunoprecipitated with antibodies against target proteins at 4°C overnight. SUMO-modified proteins were then tested by Western blotting.

### Alkaline comet assay.

The alkaline comet analyses were performed with the Comet Assay kit (R&D Systems, catalog 4250-050-K) according to the manufacturer’s instructions with minor changes. Briefly, we combined cells at 0.5 million per mL with molten low melting agarose (LMA) gel at a ratio of 1:10 (v/v) and immediately pipetted 80 μL onto the comet slice and placed it at 4°C for 30 minutes in the dark. We immersed slice into 4°C lysis buffer for 1.5 hours. Next, we immersed the slice in alkaline unwinding solution (200 mM NaOH, 1 mM EDTA, pH>13) for 20 minutes at room temperature. Finally, electrophoresis was performed in alkaline electrophoresis solution and the comet slices were stained with SYBR gold dye at room temperature for 30 minutes. The tail length was calculated by ImageJ software (NIH).

### shRNA KD and qRT-PCR.

KD of target genes by shRNAs was done as described previously (65). Scramble sequence and all the shRNAs against target genes were inserted into the pLKO.1 vector. The sequences for shRNAs are listed in Supplemental Table 2. For qRT-PCR analysis, total RNA was extracted from various cells as indicated and reverse transcribed by using kits purchased from Thermo Fisher. The primer sequences used in the qRT-PCR are listed in Supplemental Table 2.

### Cell apoptosis analysis by FACS.

0.5 × 10^6^ of the indicated cells were treated with DMSO or 20 μM cisplatin for 24 hours. After that, all the cells were collected and washed with ice-cold PBS and 1× annexin V binding buffer, respectively. Then the cells were stained by 2.5 μL anti–annexin V antibody and 1μM DAPI (final concentration) in the dark and on ice for 30 minutes. After that, the cells were subjected to flow cytometry analysis.

### Lung cancer xenograft studies.

Briefly, 2 million lung adenocarcinoma cells were subcutaneously injected into 2 flanks of each NSGS mouse. And 5 mg/kg cisplatin alone or together with 30 mg/kg STM2457 was given i.p. every 3 days when tumor volume reached approximately 100 mm^3^. Tumor volume and mouse weight measurements were taken every 4 days and 7 days, respectively. Tumor volume was calculated according to the formula [ *D* × (*d*^2^) ] /2, where *D* represents the large diameter of the tumor and *d* represents the small diameter of the tumor. Animals were individually monitored throughout the experiment.

### Analysis of mRNA m^6^A methylation by dot-blot assay.

mRNA m^6^A methylation was analyzed by dot-blot assays according to our published procedures with minor changes (13, 35). Briefly, total RNA was extracted using TRIzol reagent (Thermo Fisher, catalog 15596018), and mRNAs were separated using the Dynabeads mRNA Purification Kit (Thermo Fisher, catalog 61006). The mRNAs were denatured at 95°C for 5 minutes, followed by chilling on ice directly. Next, 400 ng mRNAs were spotted to positively charged nylon (GE healthcare), air-dried for 5 minutes, and crosslinked using a 245 nm UV cross linker. The membranes were blocked in 5% nonfat milk plus 1% BSA in PBST for 2 hours and then incubated with anti-m^6^A antibodies at 4°C overnight. After 3 times washing with PBST, the membranes were incubated with Alexa Fluor 680 goat anti-rabbit IgG secondary antibodies at room temperature for 1 hour. Membranes were subsequently scanned using image studio. Methylene blue staining was used as a loading control to make sure equal amounts of mRNAs were used for dot-blot analysis.

### m^6^A RNA IP qRT-PCR analysis.

m^6^A RNA IP (MeRIP) analyses were performed according to the published paper (66). The primer sequences used in the qRT-PCR are listed in Supplemental Table 1.

### RNA stability assay for mRNA lifetime.

All the indicated cells were treated with 5 μg per mL actinomycin D and collected at indicated time points. The total RNA was extracted by TRIzol reagent and subjected to qRT-PCR analysis. The primer sequences used in qRT-PCR are listed in Supplemental Table 2.

### m^6^A-Seq and RNA-Seq.

Total RNAs were extracted from NCI-H522 cells stably expressing empty vector and KRAS G12V by TRIzol reagent (Thermo Fisher, catalog 15596018). 10 μg of total RNAs were fragmented with RNA fragmentation buffer (Thermo Fisher, catalog AM8740), and 1 μg of RNA fragments were kept for RNA-Seq analysis. 9 μg Of RNA fragments were used for IP enrichment by using anti-m^6^A antibody (Synaptic Systems, catalog 202 003), namely for the m^6^A-seq analysis. Both the 1 μg of RNA fragments saved for the RNA-Seq analysis and the m^6^A antibody–enriched RNA fragments for m^6^A-seq analysis were rRNA depleted by using the rRNA Depletion Kit (NEB, catalog E6310L). Then the rRNA-depleted RNA fragments were used in the sequence library construction by using the NEBNext Ultra II Directional RNA Library Prep Kit for Illumina (NEB, catalog E7760L). Finally, cDNA libraries purified by using AMPure beads (Beckman Coulter, catalog A63881) were submitted to the next-generation sequencing service at the core facility of University of Florida for sequencing. All libraries were processed on a NovaSeq S4 2X150 platform (Illumina) with a paired-end 150-base pair read length, and 50 × 10^6^ reads per sample was required.

### m^6^A-Seq and RNA-Seq data analysis.

For bulk RNA-Seq analysis, bulk RNA-Seq raw sequencing reads were aligned to the human genome, hg38, and sequencing quality and alignment rate were examined using Nextflow pipeline (nf-core/rnaseq 3.12) (67). Gene expression was quantified at the gene level using Salmon. RNA-Seq libraries were then normalized using median of ratios method, and genes were tested for differential expression between the empty vector and KRAS G12V–overexpressed samples with DESeq2, version 1.36 (68). The Wald test (69) was employed to identify differentially expressed genes. For visualization, pheatmap, version 1.0.12, was used for showing differential expression between samples. The gene set enrichment test was performed using clusterProfiler, version 4.7.1 (70). KEGG (71) and Reactome (72) databases were used in GSEA. To control the false positive rate, multiple testing correction was applied using the Benjamini-Hochberg method to adjust the *P* values obtained from both the differential expression analysis and GSEA. We set a significance threshold of adjusted *P* value at 0.05 to control the false discovery rate (73).

### m^6^A-seq analysis.

m^6^A-seq raw reads were trimmed using Trim Galore, version 0.6.10. FastQC, version 0.12, was used to examine the sequencing reads quality, and low-quality reads were removed. Raw reads were aligned to human reference genome hg38, Hisat2, version 2.2.1 (74). Peaks were called using Macs2, version 2.2.7.1 (75). m^6^A-Seq libraries were normalized to RNA-Seq libraries using DiffBind, version 3.8.4 (76). Differential analysis between empty vector and KRAS G12V–overexpressed samples was performed using DESeq2, version 1.38.3 (68). For visualization, metagene plot was generated using Guitar, version 2.14.0 (77). Motif analysis was performed using homer (78). To control the false positive rate, multiple testing correction was applied using the Benjamini-Hochberg method to adjust the *P* values obtained from both the differential expression analysis and GSEA. We set a significance threshold of adjusted *P* value at 0.05 to control the false discovery rate (73).

### Statistics.

Results are presented as mean ± SD. Statistical analysis was calculated with 2-tailed Student’s *t* test or with ordinary 1-way ANOVA with Dunnett’s multiple-comparison test using GraphPad Prism 9 software. The colony-forming assay, qRT- PCR, and cell culture experiments were done with 3 technical replicates and repeated at least 3 times. *P* values equal to or less than 0.05 were considered statistically significant.

### Study approval.

All the animal studies were approved by the mouse core facility at the University of Florida.

### Data availability.

The raw and processed RNA-Seq and m^6^A-Seq data have been deposited into the NCBI’s Gene Expression Omnibus (GEO GSE268671). Values for all data points in graphs are reported in the Supporting Data Values file.

## Author contributions

ZQ and FY conceived the project. ZQ designed the research and supervised the experiments. FY, CY, SG, and RN conducted experiments and interpreted the data. ZL conducted experiments. ZS, TG, and SZ performed RNA-Seq and m^6^A-Seq data analysis. SH and LW provided NSCLC cell lines and advice for the project. FY and ZQ wrote the manuscript with inputs from all the other authors.

## Supplementary Material

Supplemental data

Unedited blot and gel images

Supplemental table 1

Supplemental table 2

Supporting data values

## Version 1 02/17/2025

In-Press Preview

## Version 2 03/17/2025

Electronic publication
