# Supplementary material for: KRAS mutants confer platinum resistance by regulating ALKBH5 posttranslational modifications in lung cancer
Source: J Clin Invest. 2025 Feb 4;135(6):e185149. doi: 10.1172/JCI185149 (PMC11910214; doi:10.1172/JCI185149)
Supplement: Supplemental data [file jci-135-185149-s062.pdf]

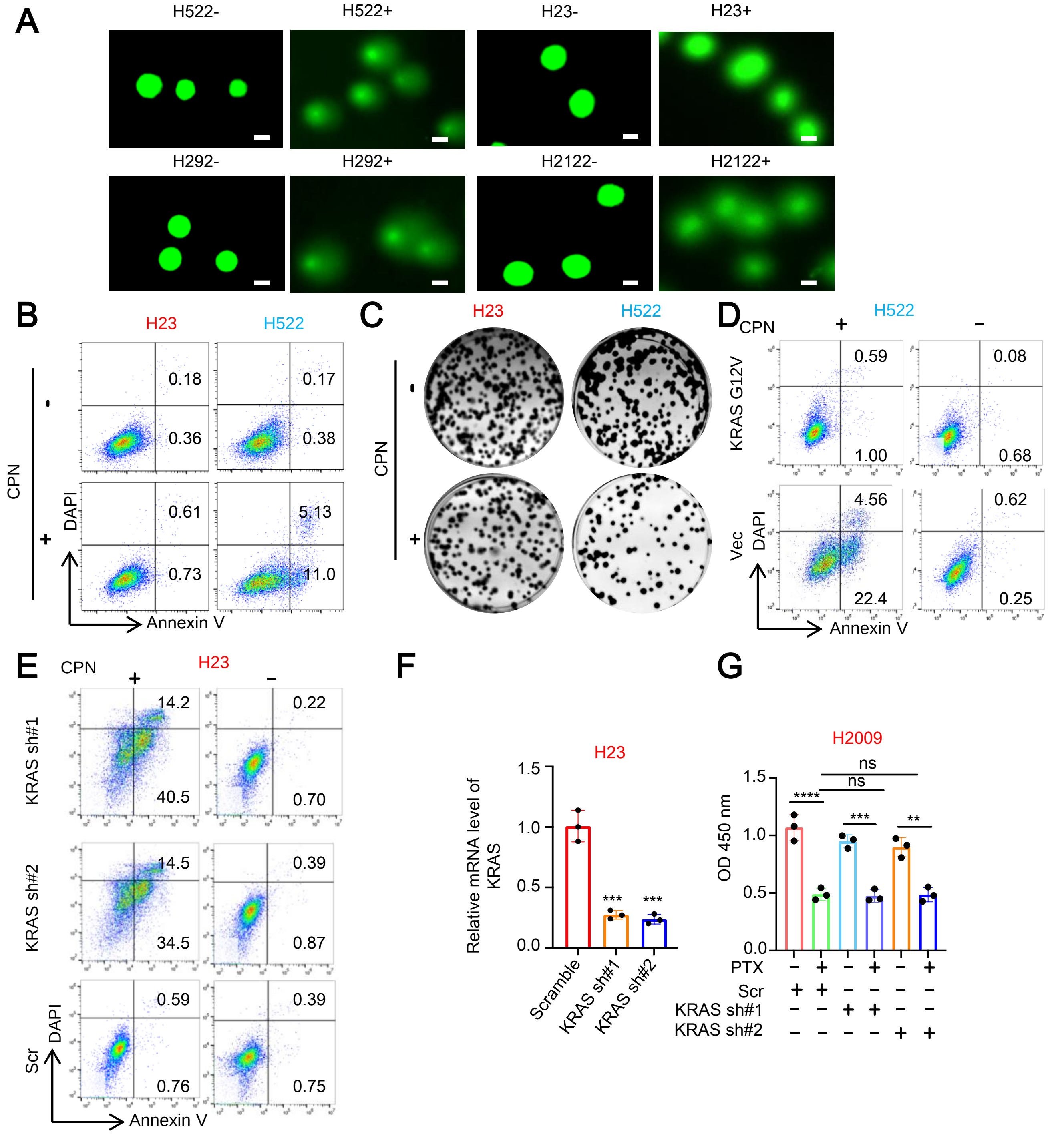

Supplemental Figure 1. Constitutively active KRAS mutant NSCLC cells are more resistant to cisplatin exposure. (**A**) Comet analysis indicating KRAS mutant cells are more resistant to cisplatin induced DNA damage. Scale bar = 124.5 μm. (**B**) Annexin V staining analysis showing that KRAS wild type NCI-H522 cells are more sensitive to cisplatin-induced cell apoptosis as compared to the KRAS mutant NCI-H23 cells. (**C**) Colony forming analysis showing that KRAS mutant NCI-H23 cells are more resistant to cisplatin as compared to the KRAS wild-type NCI-H522 cells. (**D**) Overexpression a constitutively active KRAS mutant KRAS G12V significantly facilitates cisplatin resistance of NCI-H522 cells. (**E**) KRAS knockdown markedly facilitates cisplatin sensitivity of KRAS mutant NCI-H23 cells. (**F**) RT-qPCR analysis showing the knockdown efficiency of KRAS in NCI-H23 cells. (**G**) CCK8 analysis suggests that KRAS depletion doesn't affect the paclitaxel (PTX) of KRAS mutant NCI-H2009 cells. Data are presented as mean ± SD, with ordinary 1-way ANOVA with Dunnett's multiple-comparison test used for **(F)** and **(G)**. *P < 0.05, **P < 0.01, ***P < 0.001, ****P < 0.0001.

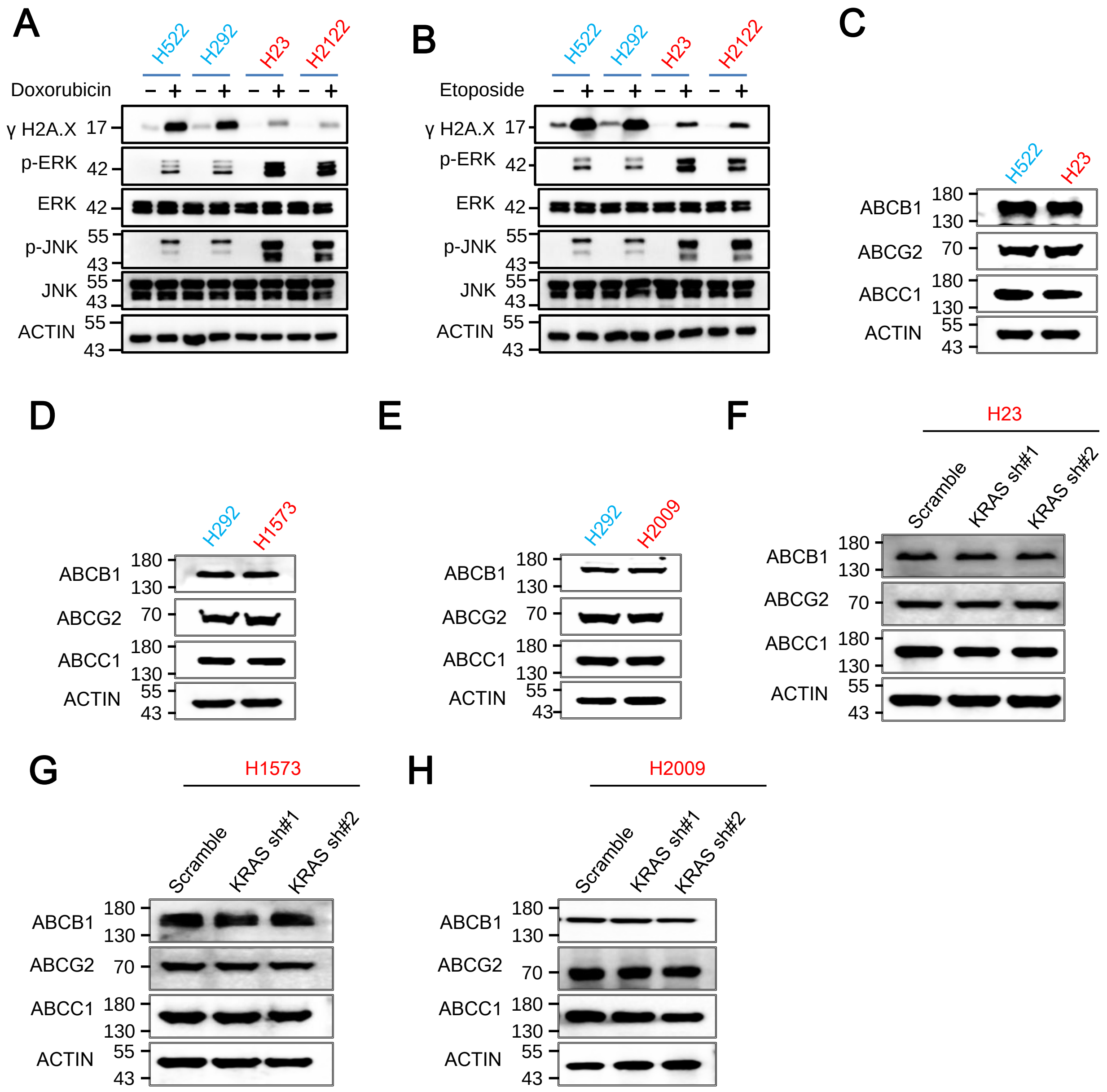

Supplemental Figure 2. KRAS depletion doesn't affect the protein levels of ABC transporter. (**A** and **B**) Western blots analysis showing the effect of Doxorubicin (A), or Etoposide (B) treatment on protein levels as indicated in KRAS wild type and mutant NSCLC cells. **(C-E**) Western blots analysis suggests that protein levels of ABC transporter including ABCB1, ABCG2, and ABCC1 are comparable in KRAS wild-type and KRAS mutant lung cancer cells. KRAS wildtype and mutant lung cancer cell lines are marked with blue and red color respectively. (**F-H**) Western blots analysis suggests that KRAS depletion can not affect protein levels of ABC transporter in KRAS mutant lung cancer cell lines.

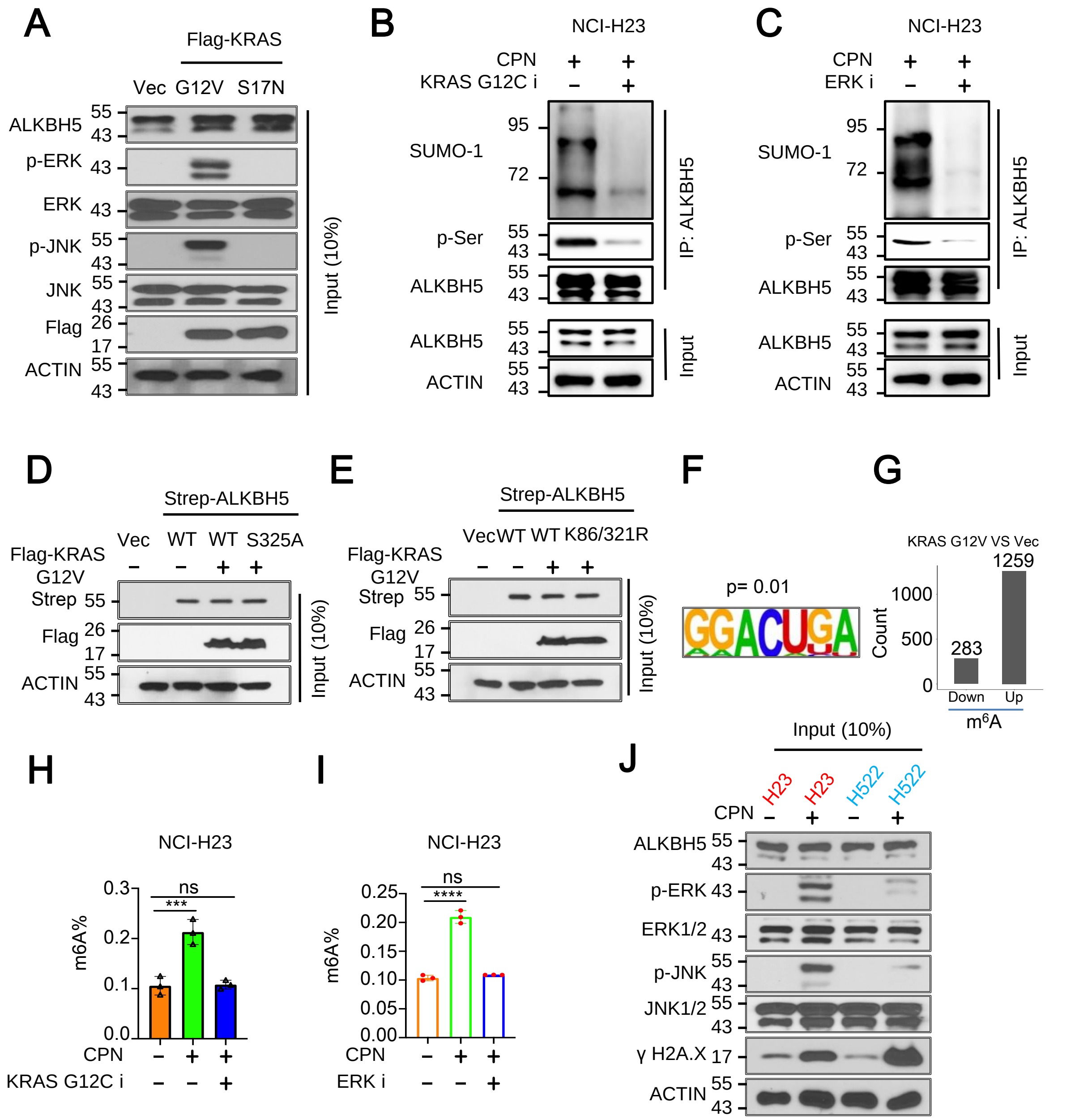

Supplemental Figure 3. Constitutively active KRAS overexpression facilitates ALKBH5 phosphorylation and SUMOylation by activating ERK/JNK signaling. (**A**) Input of Figure 2A. (**B** and **C**) Denaturing IP analysis suggests that KRAS G12C inhibitor (B), or ERK inhibitor (C) treatment blocks cisplatin-induced phosphorylation and SUMOylation of ALKBH5. (**D**) Input of Figure 2B. (**E**) Input of Figure. 2C. (**F**) Metagene analysis for the RNA RNA m^6A modification consensus motif by using the identified m^6A peaks via m^6A-seq analysis. (**G**) Histogram showing mRNA transcripts with significant m^6A modification alterations induced by KRAS G12V overexpression in NCI-H522 cells identified by m^6A-seq analysis. (**H** and **I**) m^6A quantification analysis suggests that either KRAS G12C inhibitor (H), or ERK inhibitor treatment blocks cisplatin-induced RNA m^6A methylation in NCI-H23 cells. (**J**) Input of Figure 2M. Data are presented as mean ± SD, with ordinary 1-way ANOVA with Dunnett's multiple-comparison test used for **(H)** and **(I)**. *$P < 0.05$, **$P < 0.01$, ***$P < 0.001$, ****$P < 0.0001$.

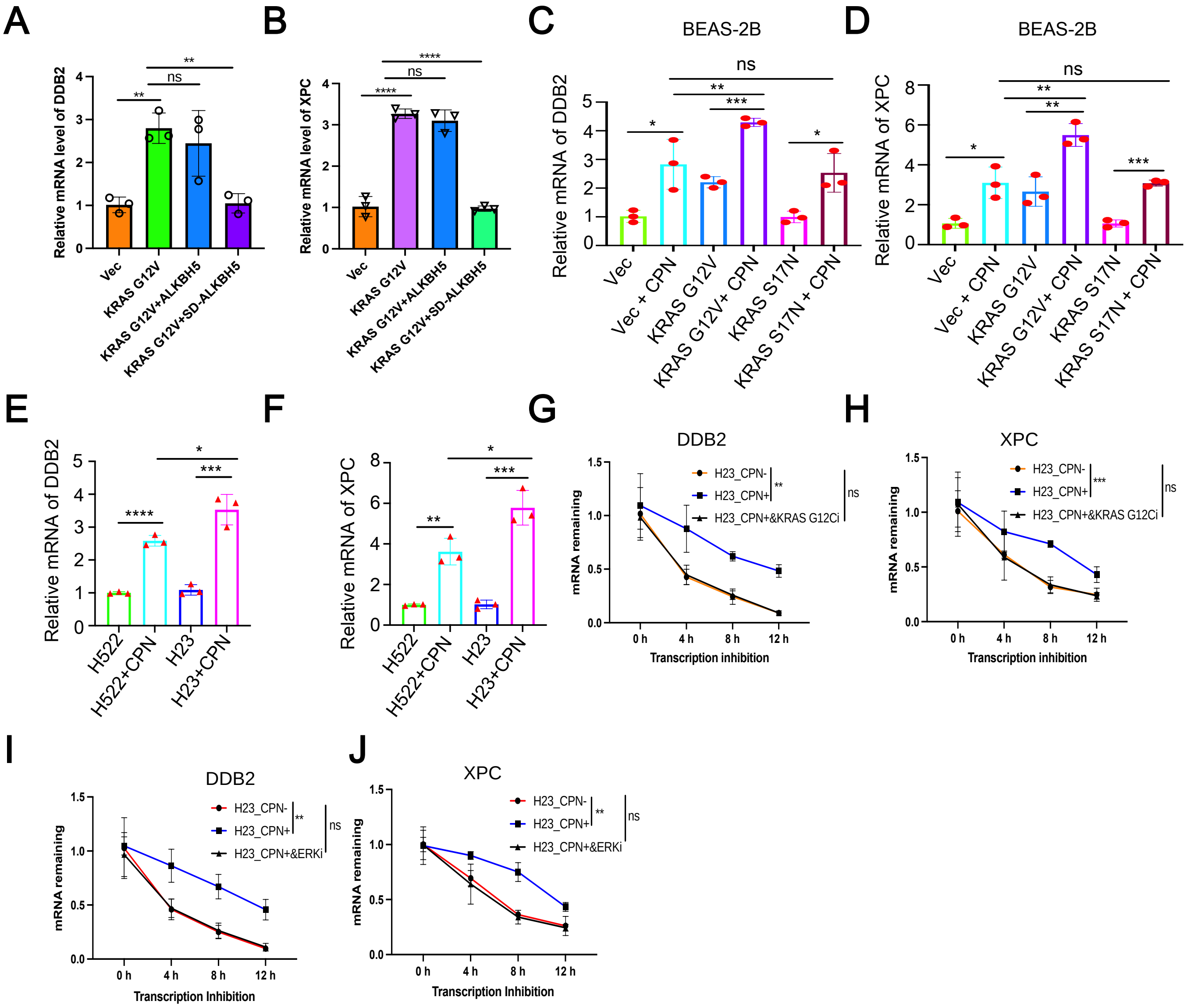

Supplemental Figure 4. Constitutively active KRAS overexpression facilitates cisplatin-induced upregulation of DNA repair related genes. (**A** and **B**) RT-qPCR analysis showing the effect of wild-type or SUMOylation-deficient mutant ALKBH5 overexpression on KRAS G12V-induced upregulation of DDB2 (A) and XPC (B). (**C** and **D**) RT-qPCR analysis of the KRAS downstream target gens related to DNA damage repair in the cells as indicated. (**E** and **F**) Gene expressions of KRAS downstream target genes related to DNA damage repair are more easily induced in KRAS mutation harboring NSCLC cells than the wild-type K-RAS carrying cells. (**G-J**) mRNA half-life analyses suggest that cisplatin-induced mRNA stabilization of DDB2 and XPC could be blocked by either KRAS G12C inhibitor (G and H), or ERK inhibitor (I and J) treatment. All data are presented as mean ± SD, with ordinary 1-way ANOVA with Dunnett's multiple-comparison test. *$P < 0.05$, **$P < 0.01$, ***$P < 0.001$, ****$P < 0.0001$.

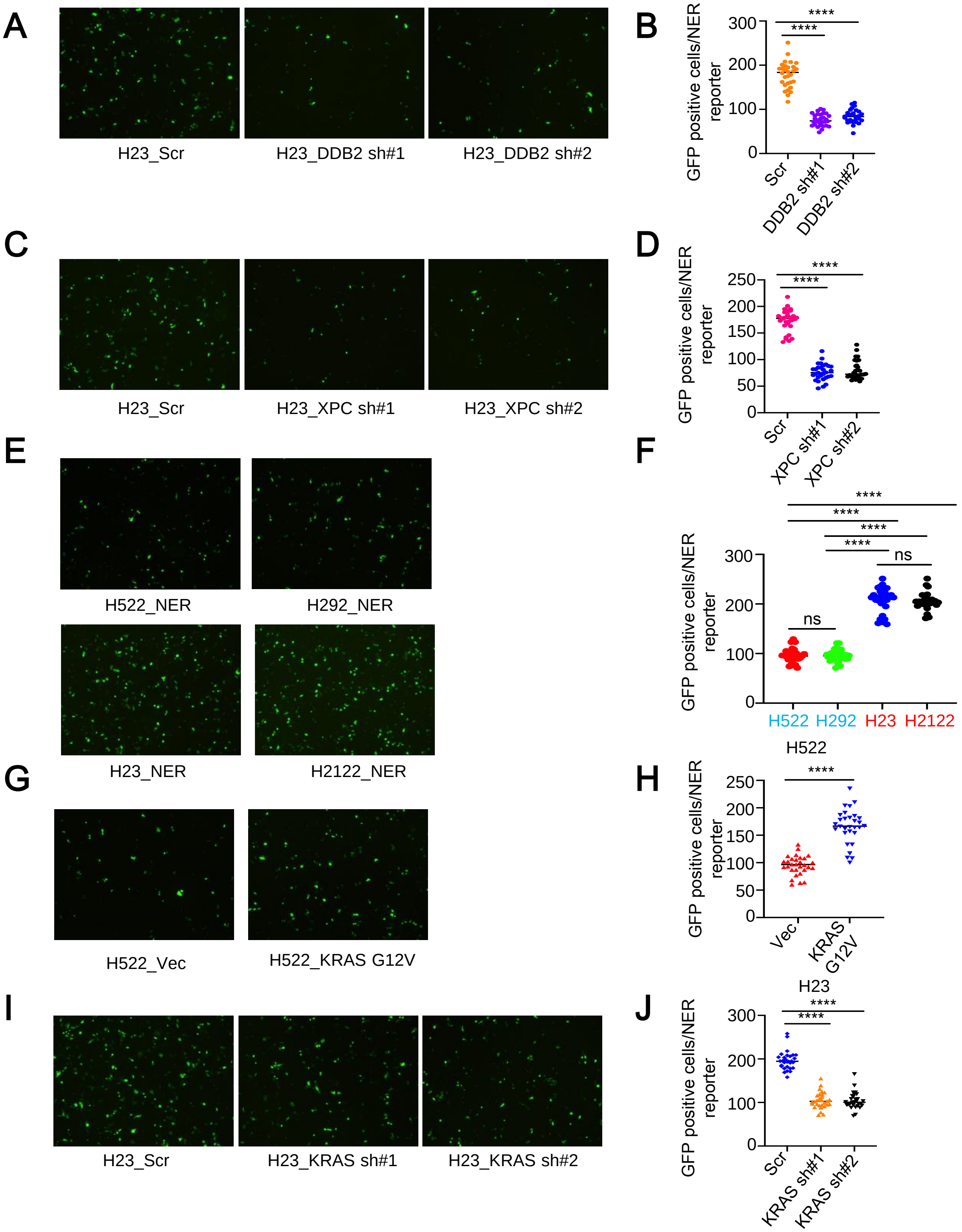

Supplemental Figure 5. KRAS mutation positively regulates nucleotide excision repair in NSCLC cells. (**A-D**) nucleotide excision repair (NER) reporter analyses suggest that either DDB2 (A and B), or XPC (C and D) knockdown significantly inhibits NER capacity in NCI-H23 cells. (**E** and **F**) NER reporter analysis suggests that the NER capacity in KRAS-mutant lung cancer cells, including H23 and H2122 is higher than that in KRAS wild-type cells such as H522 and H292. (**G** and **H**) NER reporter analysis suggests NER capacity in H522 cells could be significantly enhanced by overexpressing KRAS G12V. (**I** and **J**) NER reporter analysis suggests that KRAS knockdown markedly inhibits NER capacity in H23 cells. Data are presented as mean ± SD, with ordinary 1-way ANOVA with Dunnett's multiple-comparison test used for **(B,D,F,J)** and and 2-tailed Student's *t* test for **(H)**. **P* < 0.05, ***P* < 0.01, ****P* < 0.001, *****P* < 0.0001.

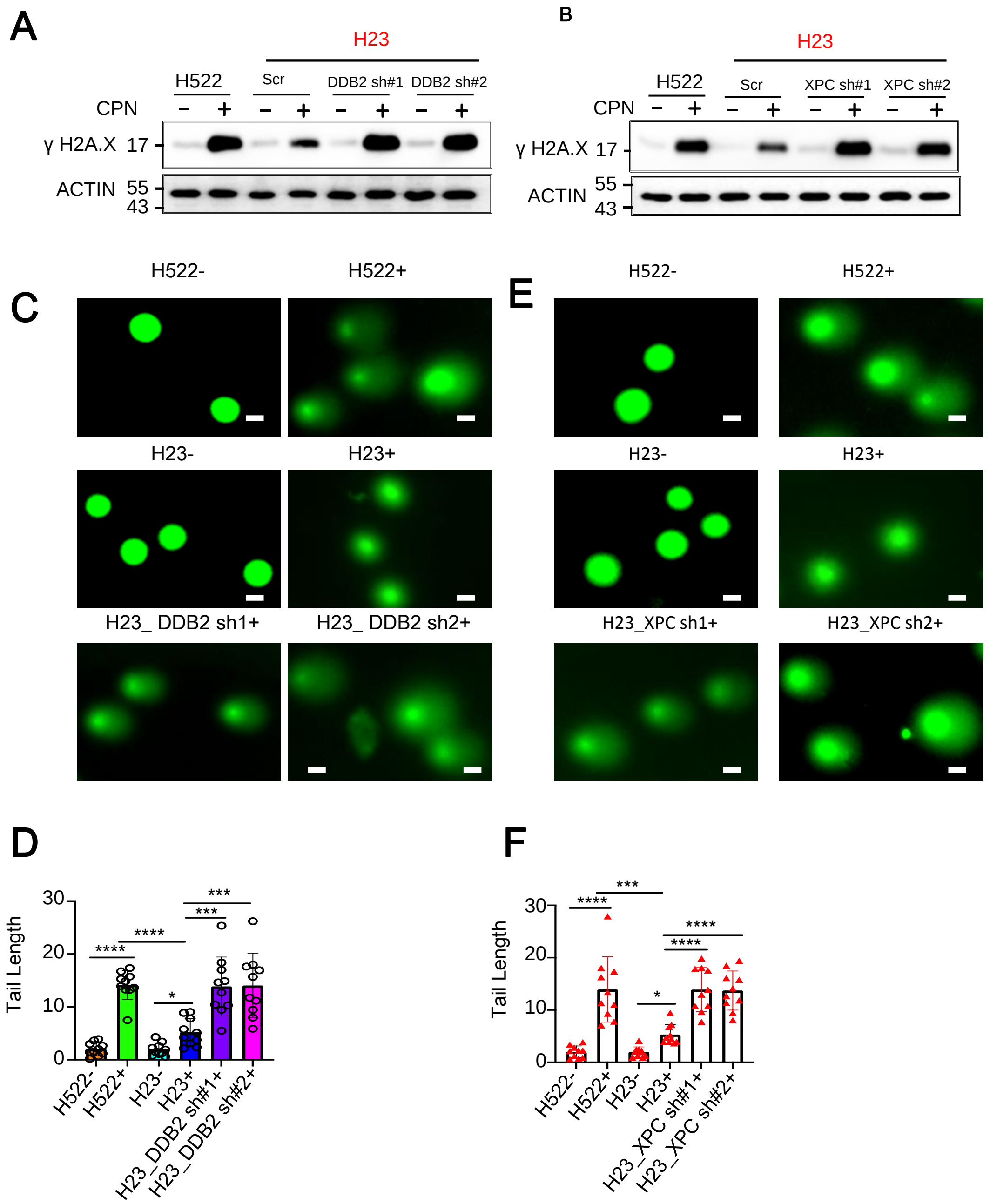

Supplemental Figure 6. KRAS mutation confers platinum resistance in NSCLC cells by modulating NER components, including DDB2 and XPC. (**A** and **B**) Western blots analysis suggests that either DDB2 (A), or XPC (B) knockdown significantly sensitizes H23 cells to cisplatin-induced DNA damage. (**C-F**) Comet analyses indicate that either DDB2 (C and D), or XPC (E and F) knockdown significantly sensitizes H23 cells to cisplatin-induced DNA damage. Scale bar = 124.5 μm. Data are presented as mean ± SD, with ordinary 1-way ANOVA with Dunnett's multiple-comparison test used for **(D)** and **(F)**. **P* < 0.05, ***P* < 0.01, ****P* < 0.001, *****P* < 0.0001.

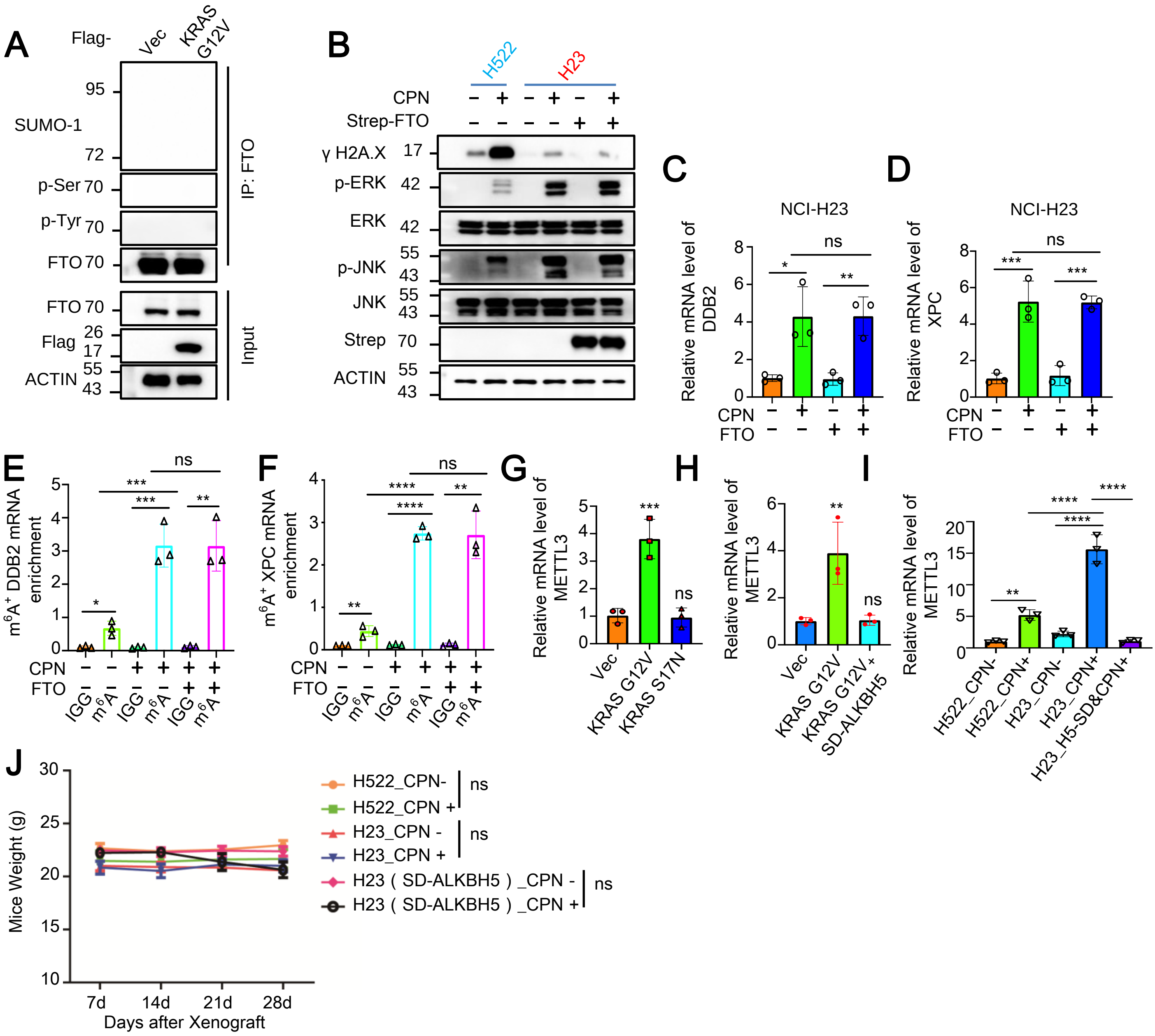

Supplemental Figure 7. KRAS mutation-mediated cisplatin resistance by regulating SUMOylation of ALKBH5. (**A**) Denaturing IP analysis suggests that both phosphorylation and SUMOylation of FTO can not be regulated by KRAS G12V overexpression. (**B**) Western blots analysis showing the effect of FTO overexpression on cisplatin sensitivity in NCI-H23 cells. (**C** and **D**) RT-qPCR analyses indicate that cisplatin-induced upregulation of DDB2 and XPC can not be blocked by FTO overexpression. (**E** and **F**) MeRIP analyses suggest that FTO overexpression doesn't affects cisplatin-induced m^6A methylation on DDB2 and XPC transcripts. (**G**) RT-qPCR analysis suggests that KRAS G12V overexpression induces METTL3 transcription. (**H**) RT-qPCR analysis indicates that KRAS G12V overexpression-induced METTL3 upregulation could be blocked by overexpressing SUMOylation-deficient mutant ALKBH5 (SD-ALKBH5). (**I**) RT-qPCR analyses suggest that mRNA levels of METTL3 are more significantly induced by cisplatin in NCI-H23 cells as compared to NCI-H522 cells, and cisplatin-induced upregulation of METTL3 could be blocked by overexpressing SUMOylation-deficient mutant ALKBH5. (**J**) Mice weight were neither affected by cisplatin injection nor tumor growth. Mice weight were measured every 7 days after lung cancer cells injection. Data are presented as mean ± SD, with ordinary 1-way ANOVA with Dunnett's multiple-comparison test used for **(C-J)**. $*P < 0.05$, $**P < 0.01$, $***P < 0.001$, $****P < 0.0001$.

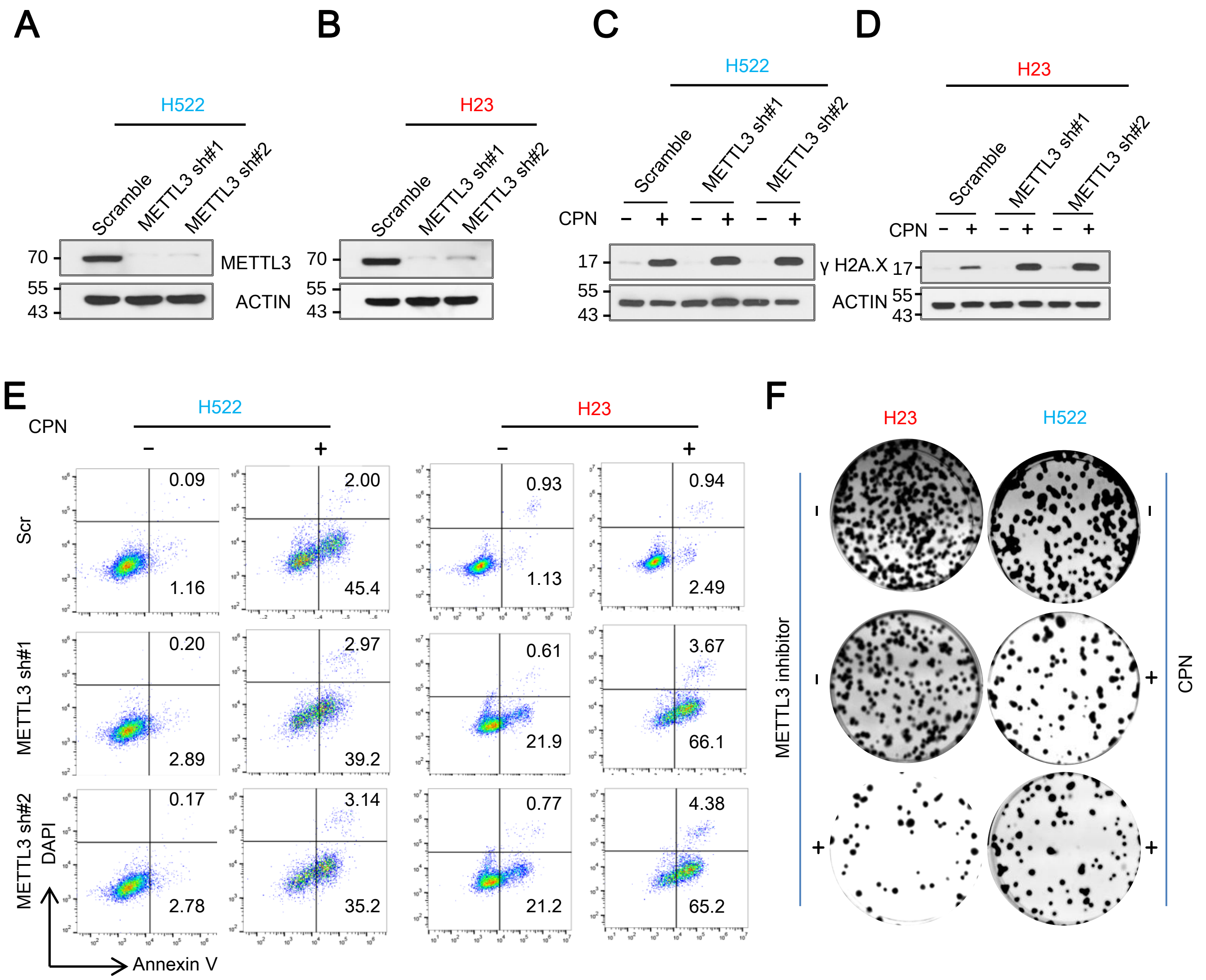

Supplemental Figure 8. METTL3 depletion sensitizes KRAS mutation harboring NCI-H23 cells to cisplatin-induced DNA damage and cell apoptosis. (**A** and **B**) Western blots analysis showing the knockdown efficiency of METTL3 in wild-type KRAS carrying NCI-H522 cells and KRAS constitutively active mutation harboring NCI-H23 cells, respectively. (**C** and **D**) Western blot analysis showing the effect of METTL3 knockdown on the cisplatin-induced DNA damage in NCI-H522 and NCI-H23 cells, respectively. (**E**) Annexin V staining analysis showing the effect of METTL3 knockdown on cell apoptosis of NCI-H522 (KRAS wild-type) and NCI-H23 (KRAS mutant) respectively. (**F**) Colony forming analysis were performed by using the cells as indicated.
