# Supplementary material for: KRAS mutants confer platinum resistance by regulating ALKBH5 posttranslational modifications in lung cancer
Source: J Clin Invest. 2025 Feb 4;135(6):e185149. doi: 10.1172/JCI185149 (PMC11910214; doi:10.1172/JCI185149)
Supplement: Unedited blot and gel images [file jci-135-185149-s063.pdf]

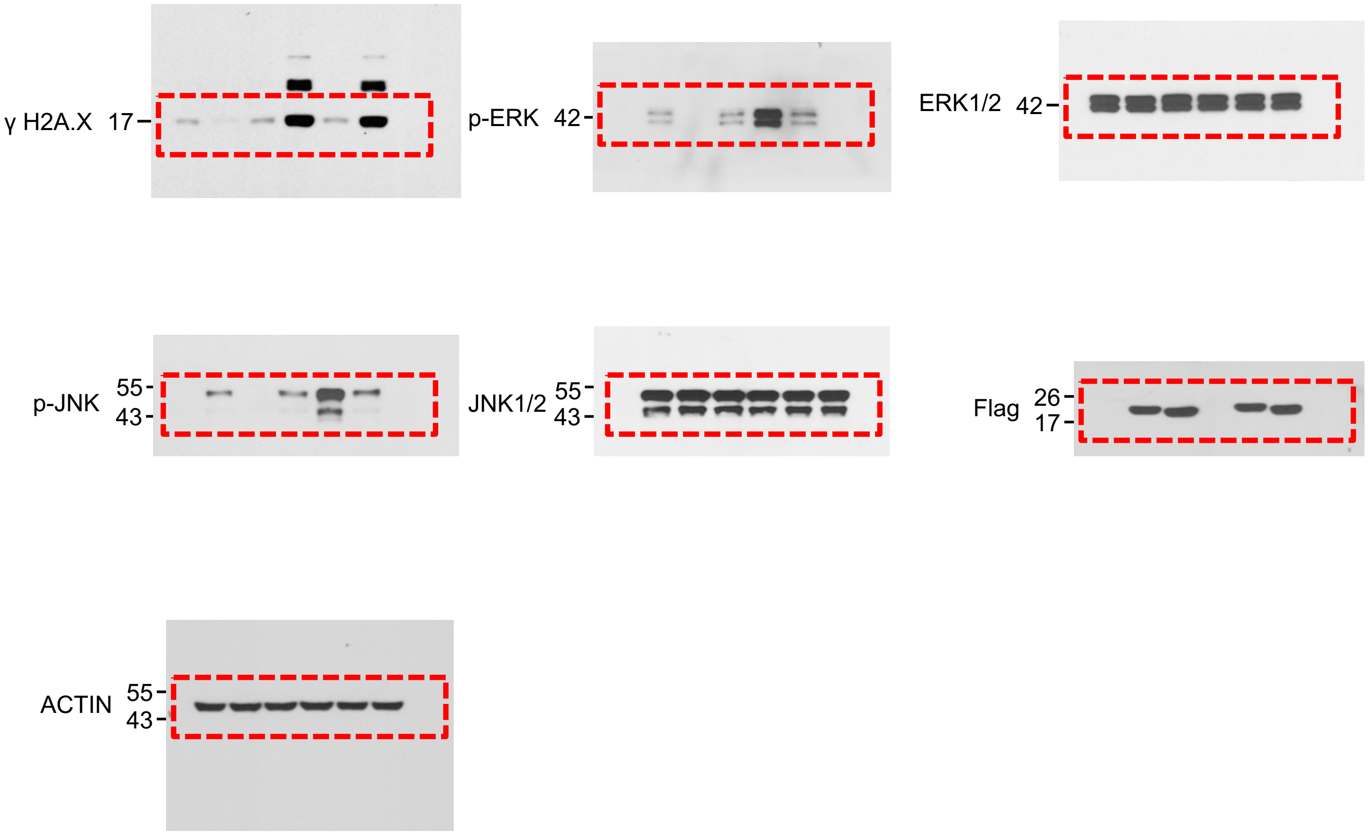

Full unedited Western blot. Red boxes indicate the images used in Figure 1A.

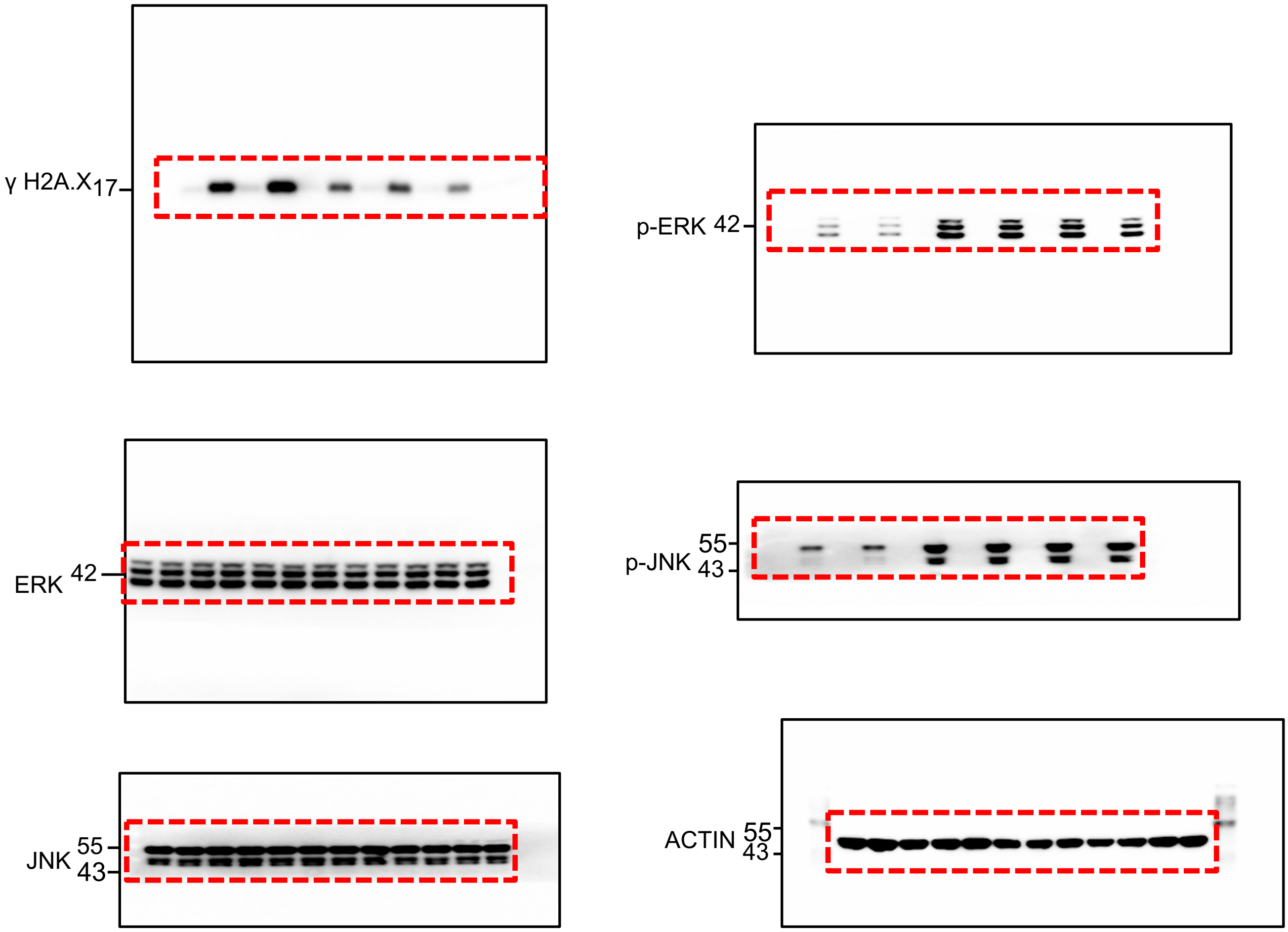

Full unedited Western blot. Red boxes indicate the images used in Figure 1B.

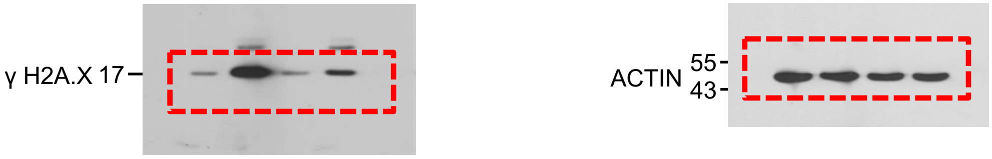

Full unedited Western blot. Red boxes indicate the images used in Figure 1G.

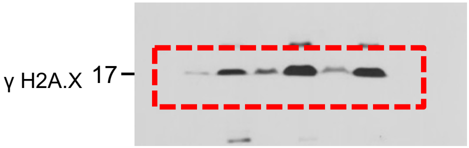

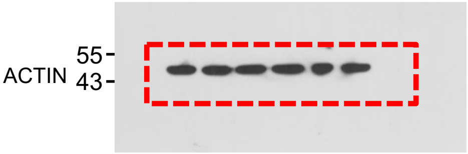

Full unedited Western blot. Red boxes indicate the images used in Figure 1I.

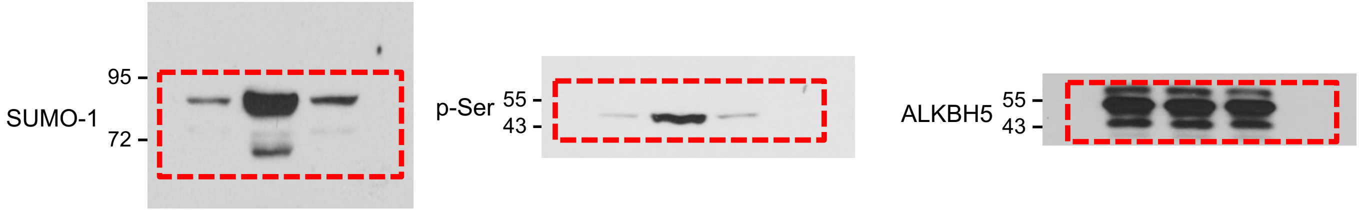

Full unedited Western blot. Red boxes indicate the images used in Figure 2A.

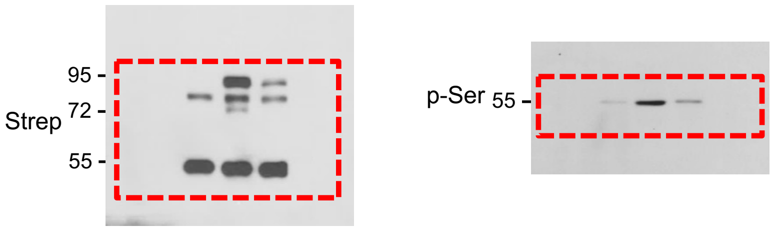

Full unedited Western blot. Red boxes indicate the images used in Figure 2B.

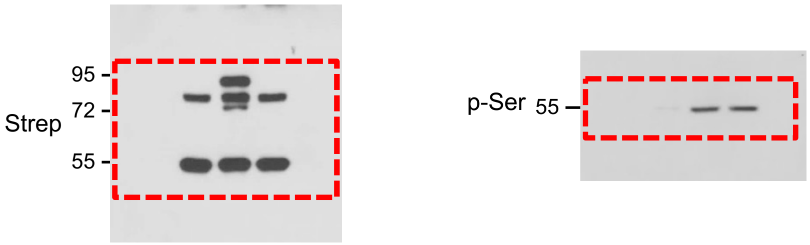

Full unedited Western blot. Red boxes indicate the images used in Figure 2C.

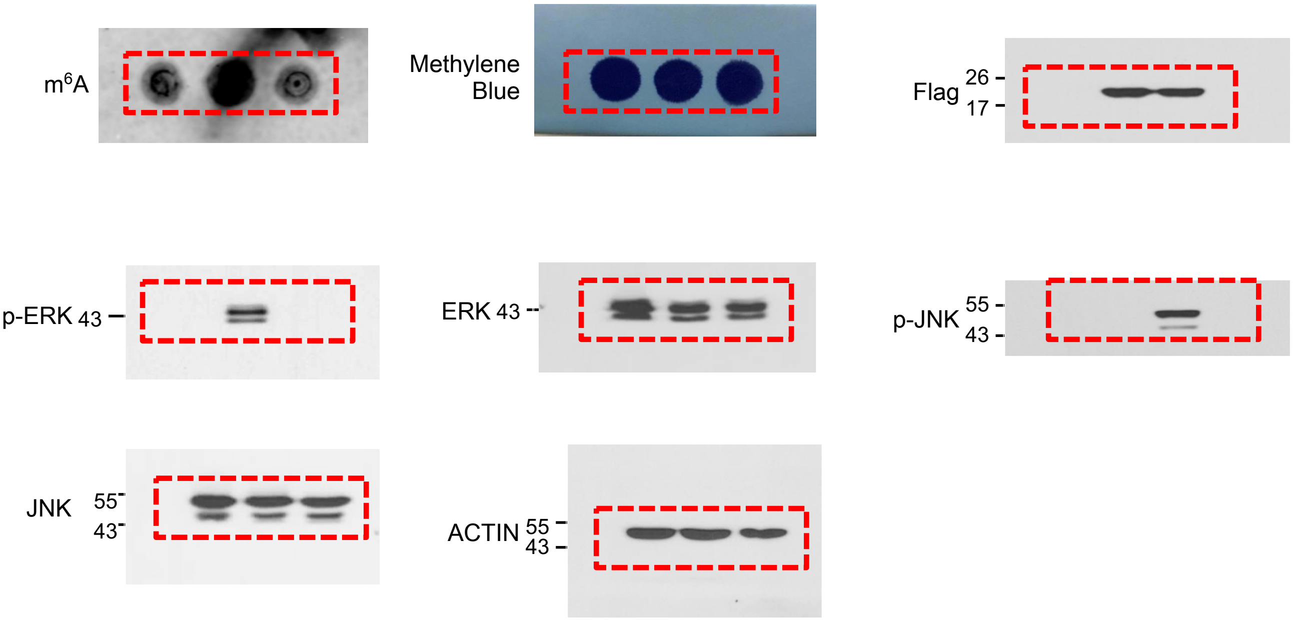

Full unedited Dot blot and Western blot. Red boxes indicate the images used in Figure 2D.

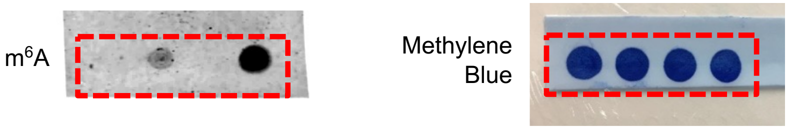

Full unedited Dot blot. Red boxes indicate the images used in Figure 2H.

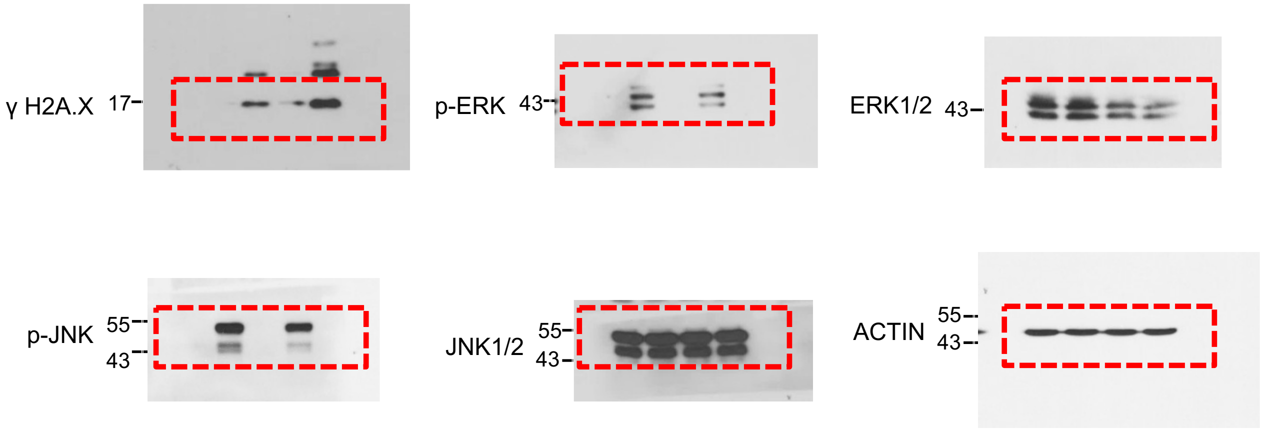

Full unedited Western blot. Red boxes indicate the images used in Figure 2I.

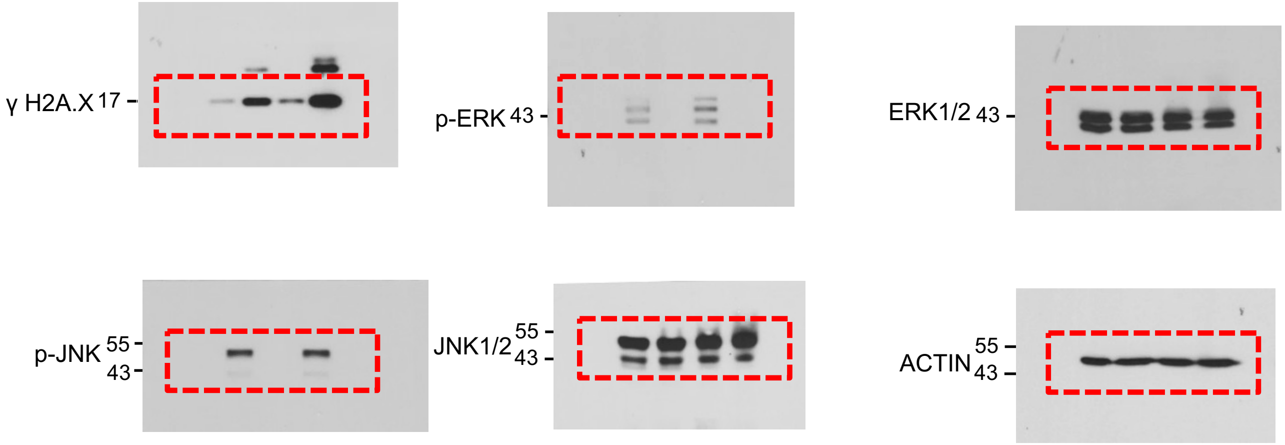

Full unedited Dot blot. Red boxes indicate the images used in Figure 2J.

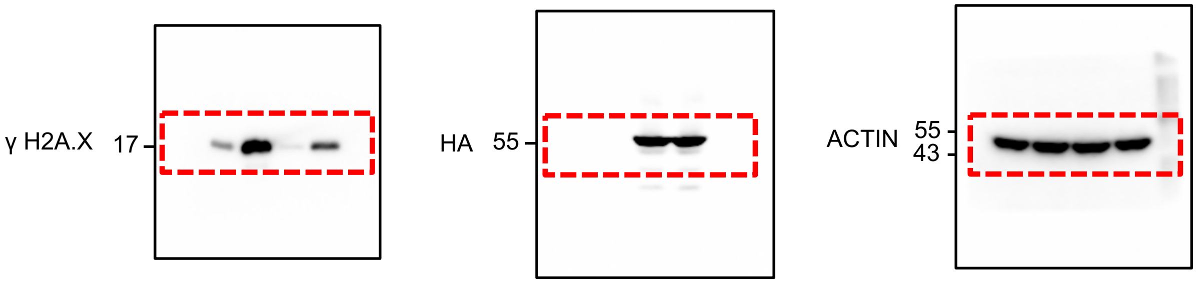

Full unedited Western blot. Red boxes indicate the images used in Figure 2K.

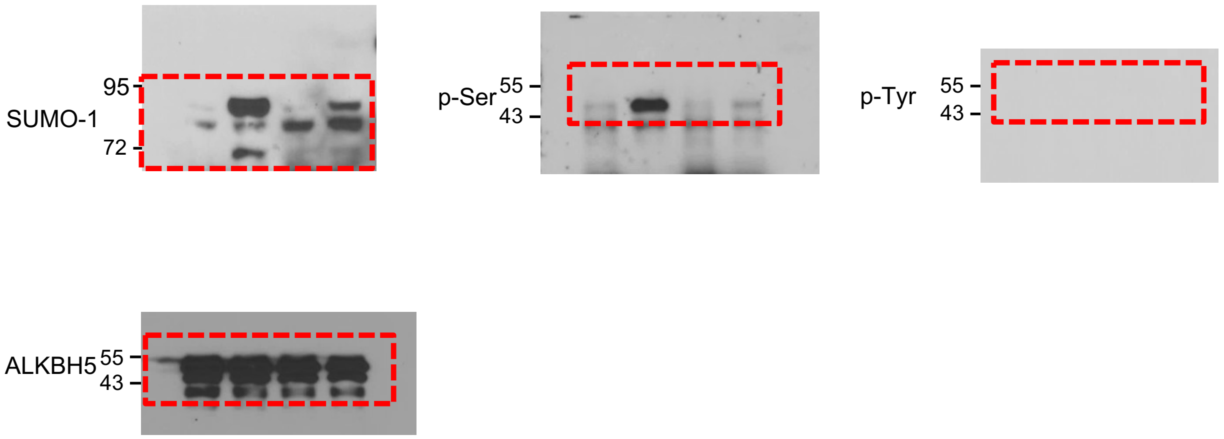

Full unedited Western blot. Red boxes indicate the images used in Figure 2L.

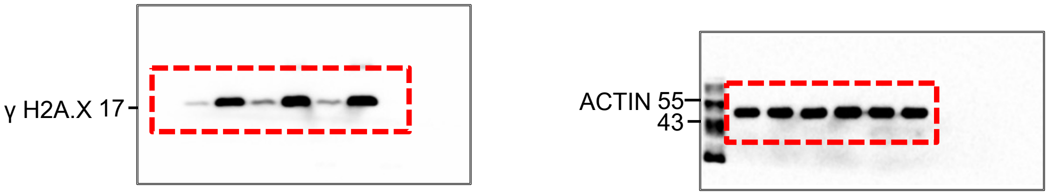

Full unedited Western blot. Red boxes indicate the images used in Figure 3A.

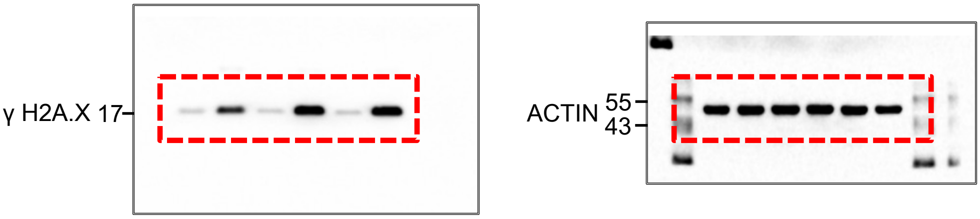

Full unedited Western blot. Red boxes indicate the images used in Figure 3C.

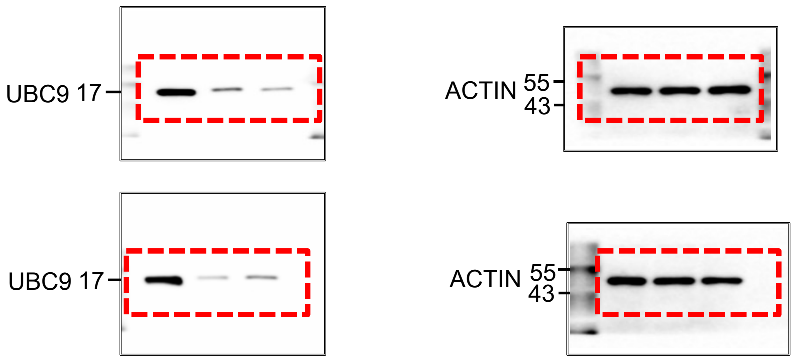

Full unedited Western blot. Red boxes indicate the images used in Figure 3E.

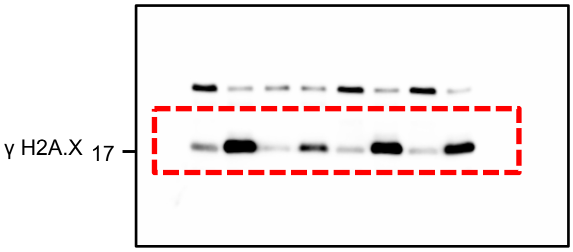

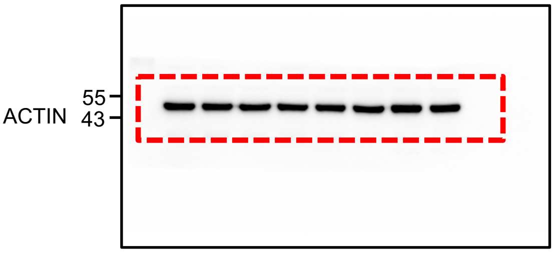

Full unedited Western blot. Red boxes indicate the images used in Figure 5K.

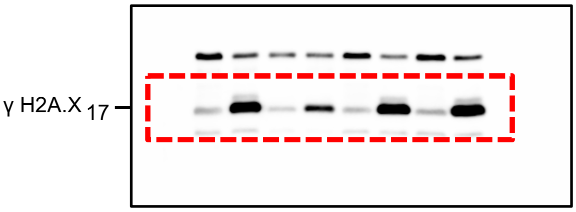

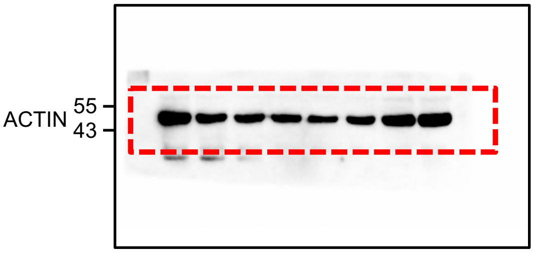

Full unedited Western blot. Red boxes indicate the images used in Figure 5L.

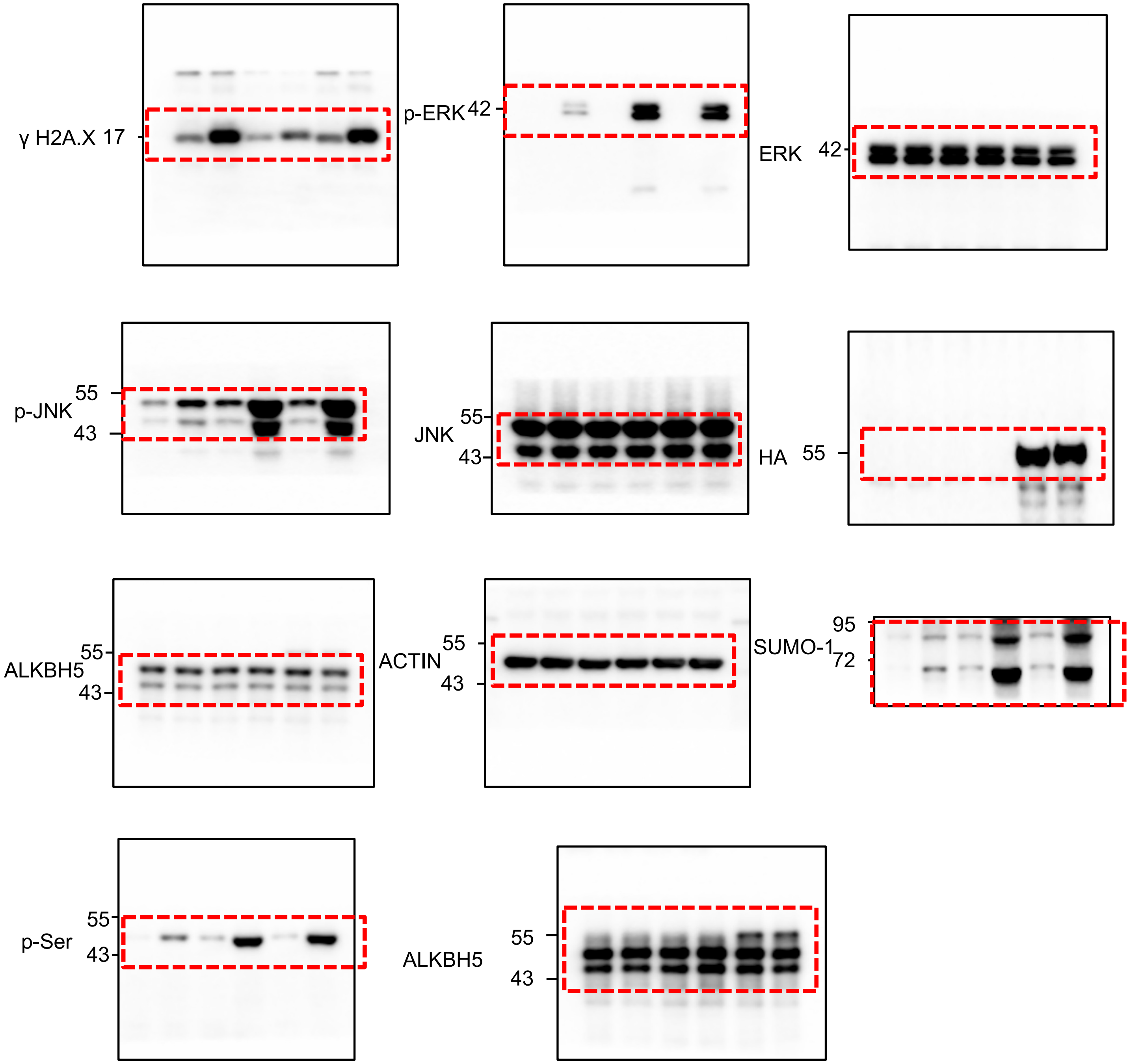

Full unedited Western blot. Red boxes indicate the images used in Figure 6D.

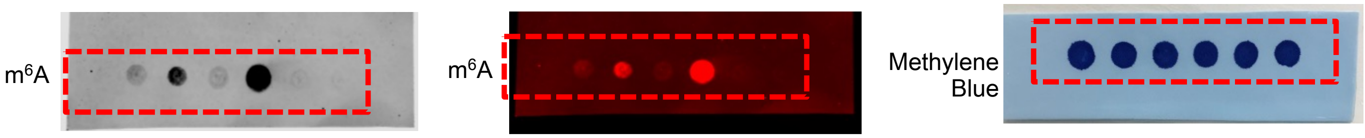

Full unedited Dot blot. Red boxes indicate the images used in Figure 6E.

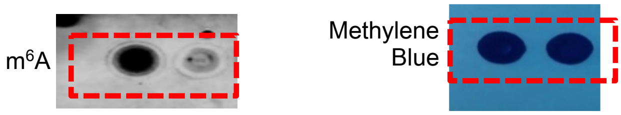

Full unedited Dot blot. Red boxes indicate the images used in Figure 7B.

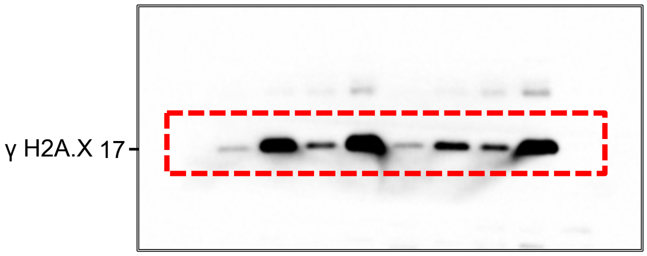

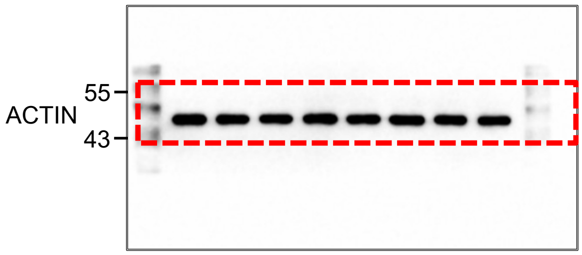

Full unedited Western blot. Red boxes indicate the images used in Figure 7C.

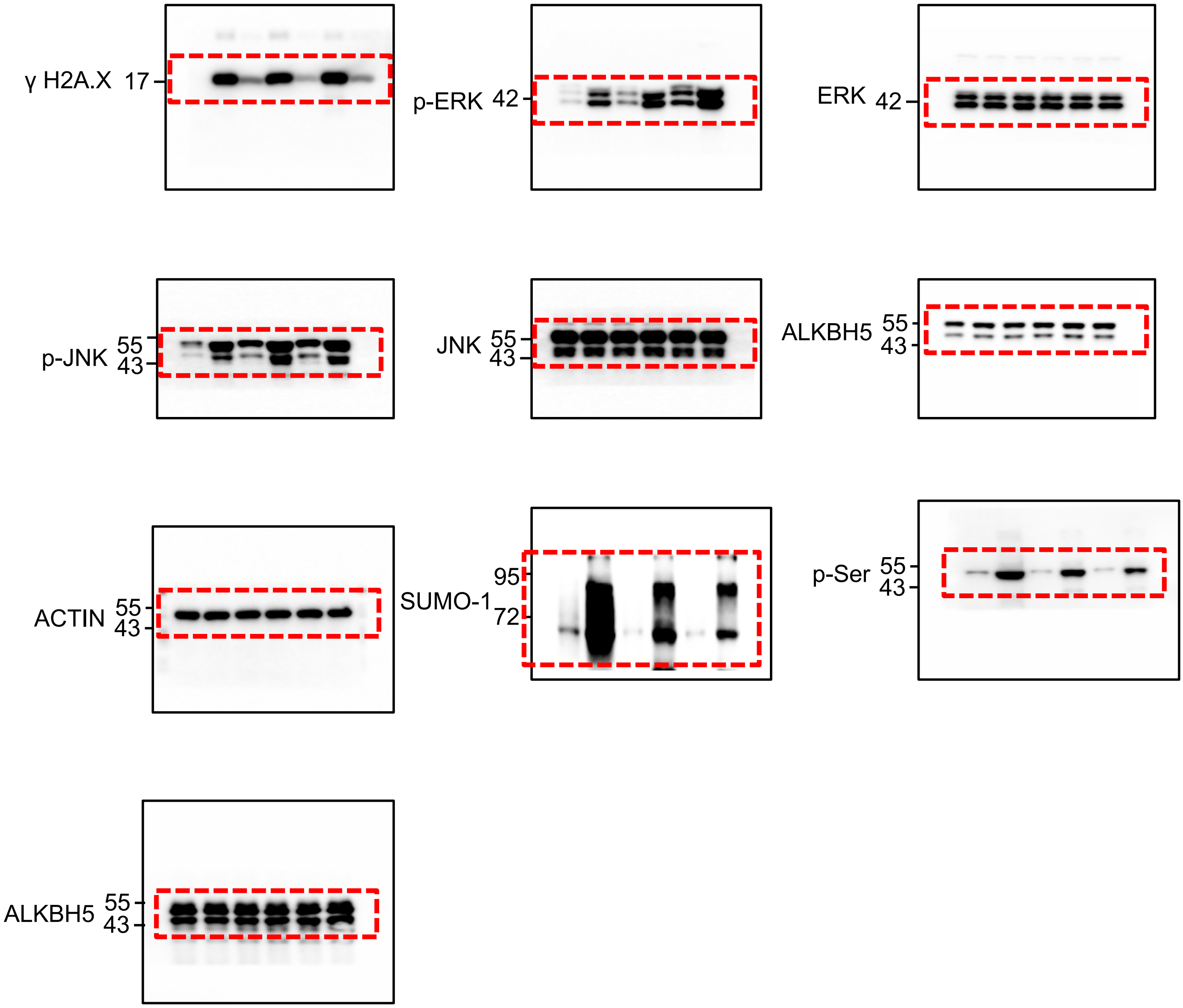

Full unedited Western blot. Red boxes indicate the images used in Figure 8A.

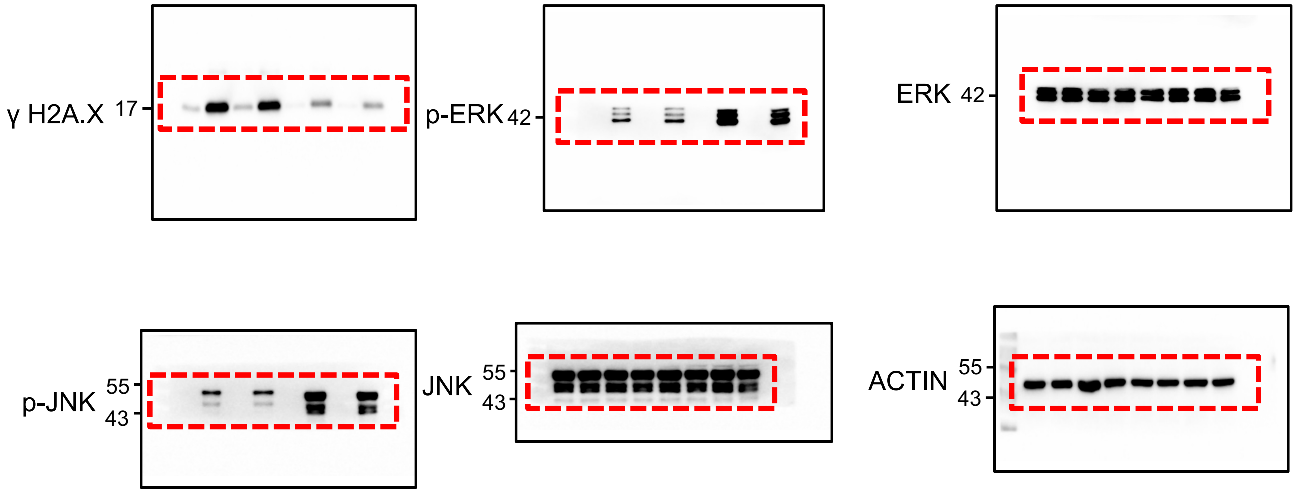

Full unedited Western blot. Red boxes indicate the images used in Supplemental Figure 2A.

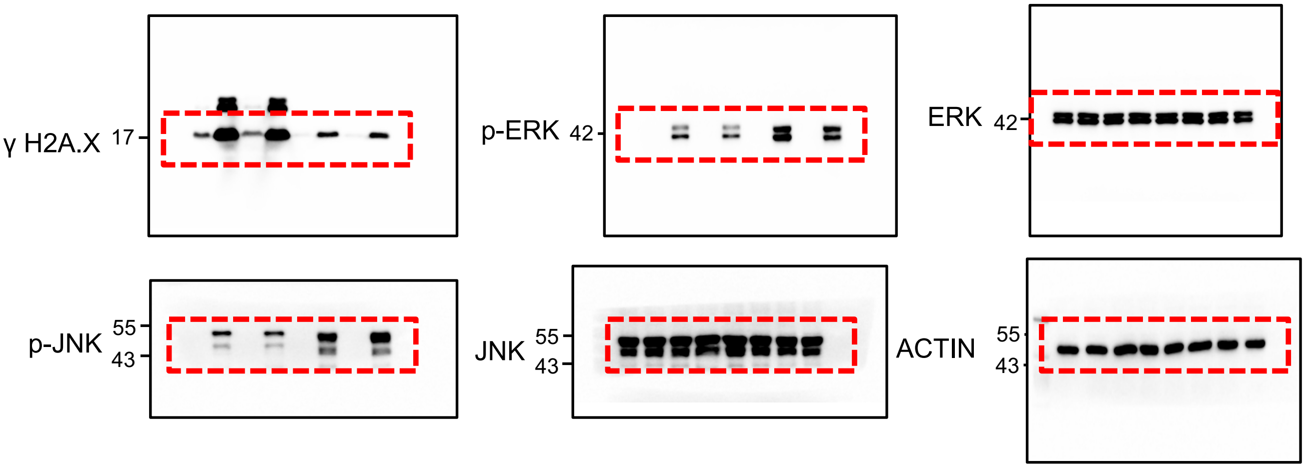

Full unedited Western blot. Red boxes indicate the images used in Supplemental Figure 2B.

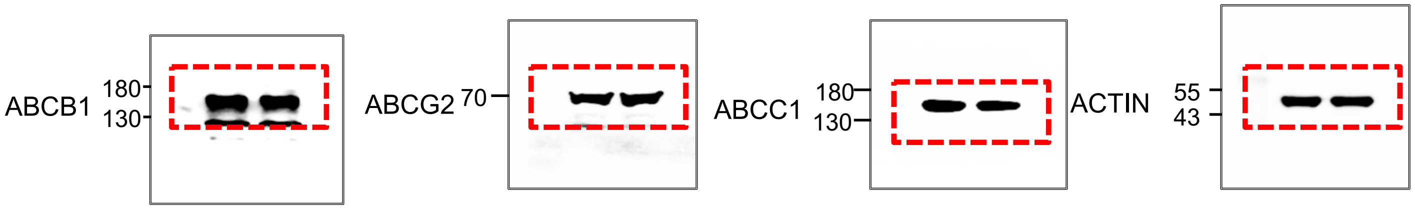

Full unedited Western blot. Red boxes indicate the images used in Supplemental Figure 2C.

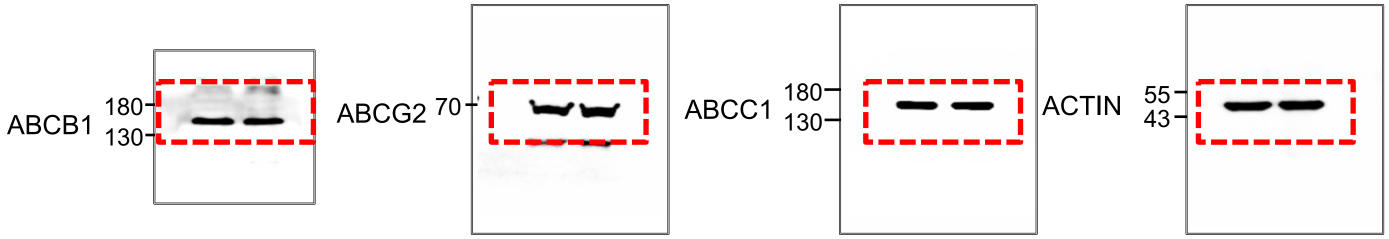

Full unedited Western blot. Red boxes indicate the images used in Supplemental Figure 2D.

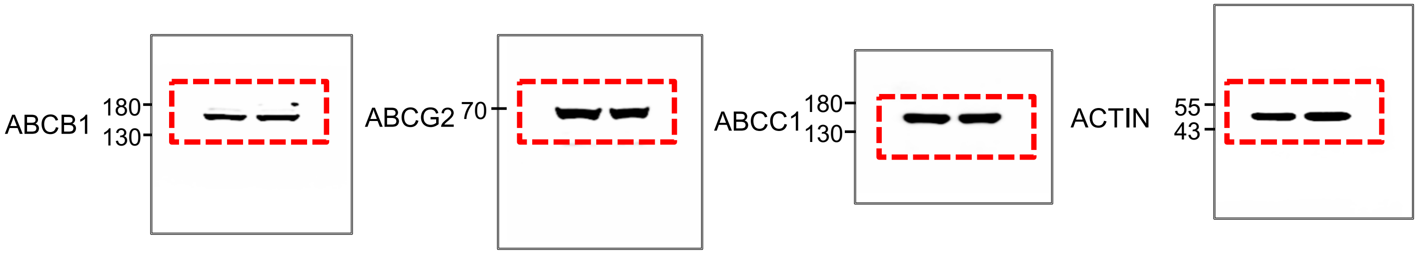

Full unedited Western blot. Red boxes indicate the images used in Supplemental Figure 2E.

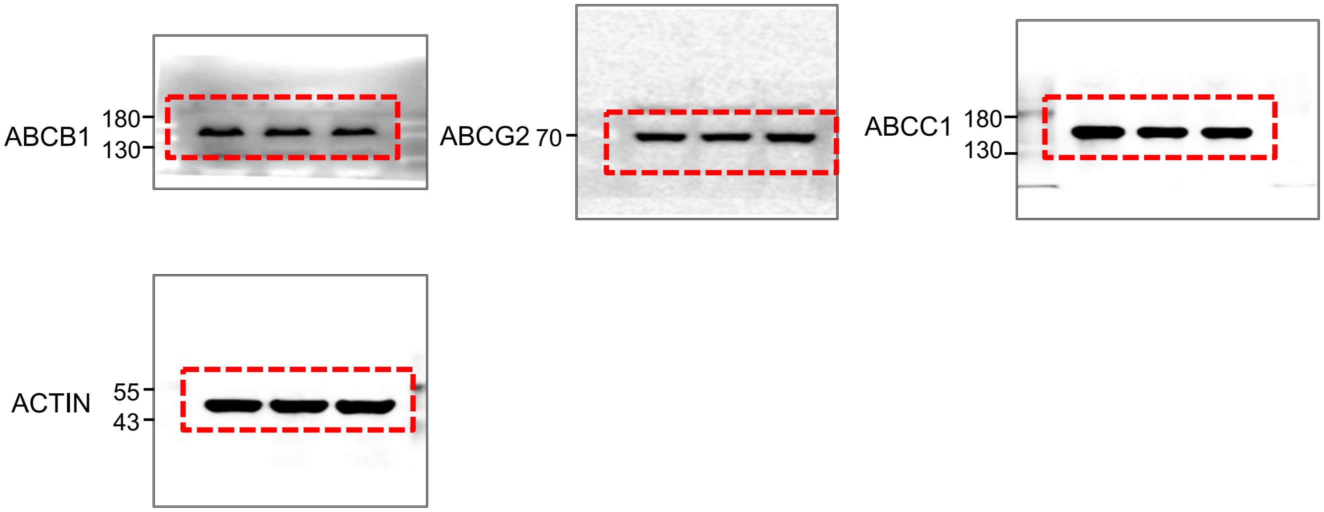

Full unedited Western blot. Red boxes indicate the images used in Supplemental Figure 2F.

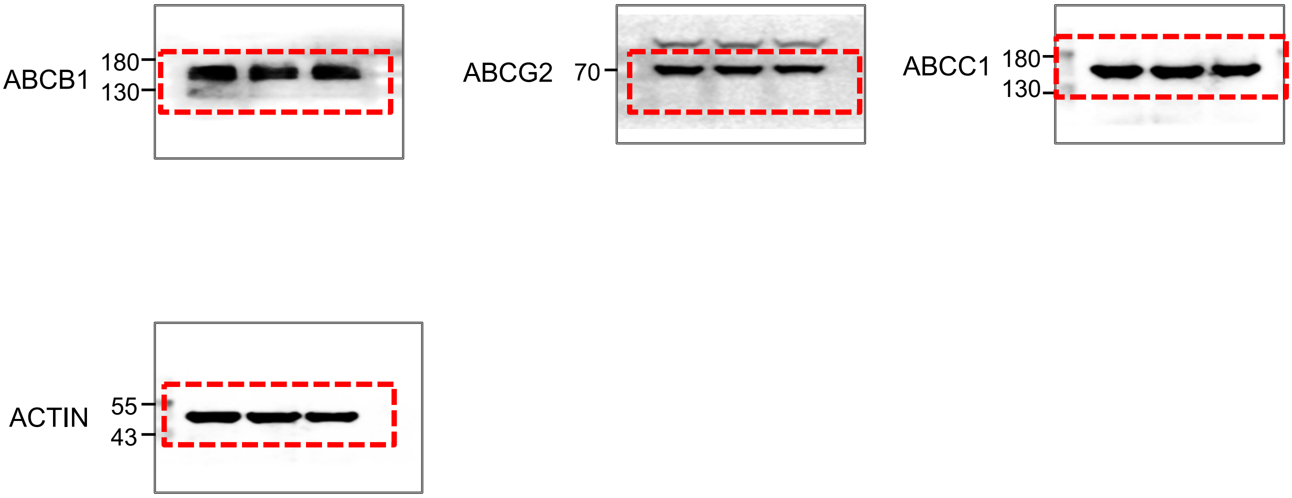

Full unedited Western blot. Red boxes indicate the images used in Supplemental Figure 2G.

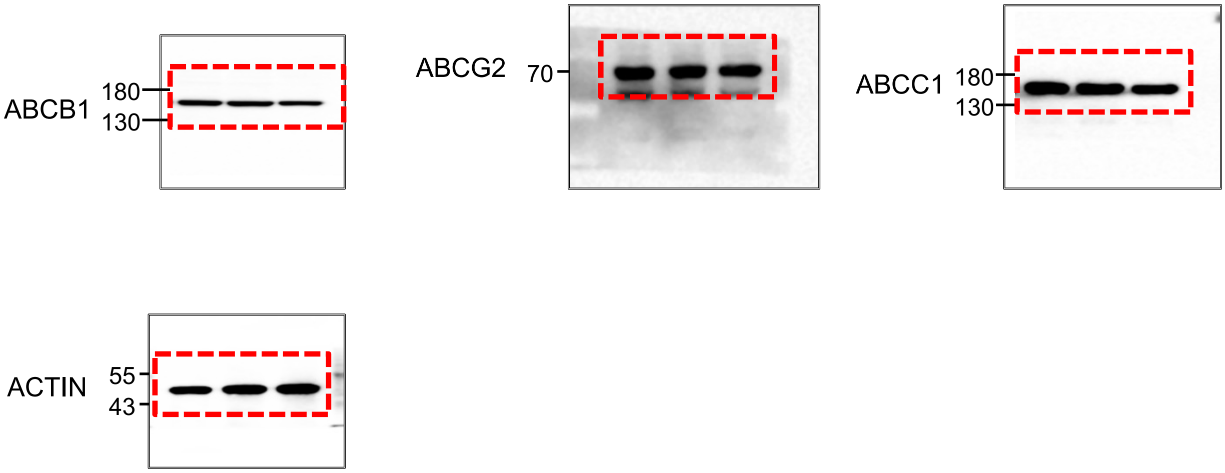

Full unedited Western blot. Red boxes indicate the images used in Supplemental Figure 2H.

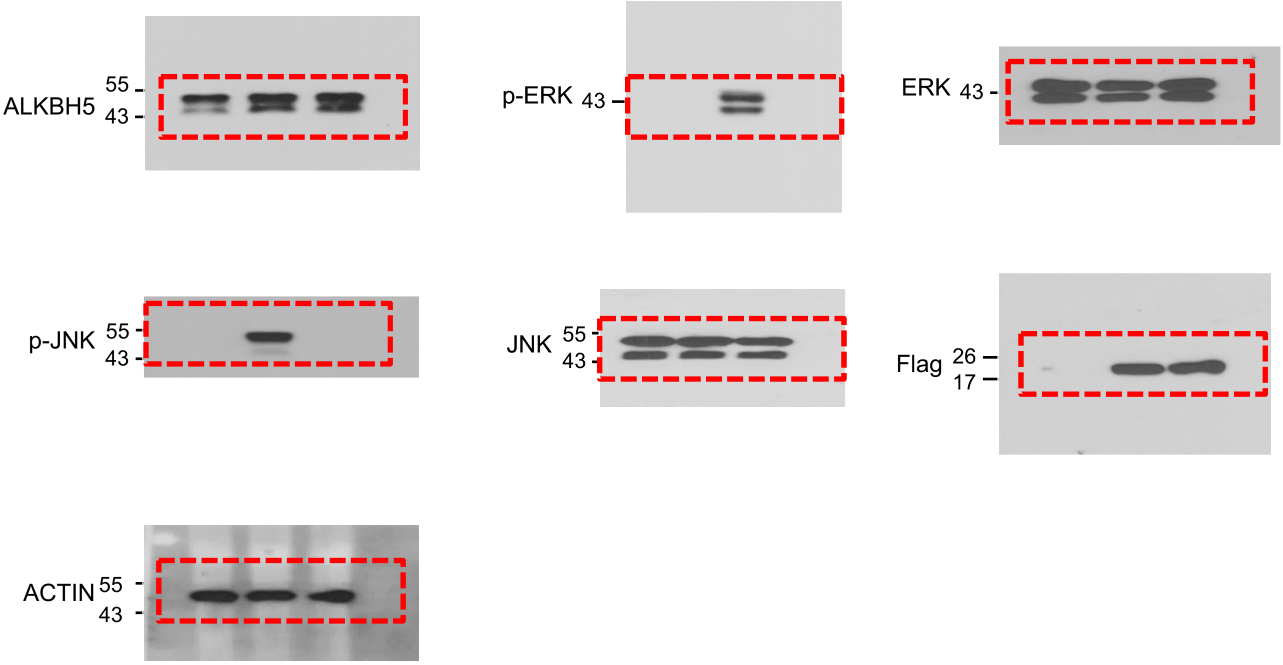

Full unedited Western blot. Red boxes indicate the images used in Supplemental Figure 3A.

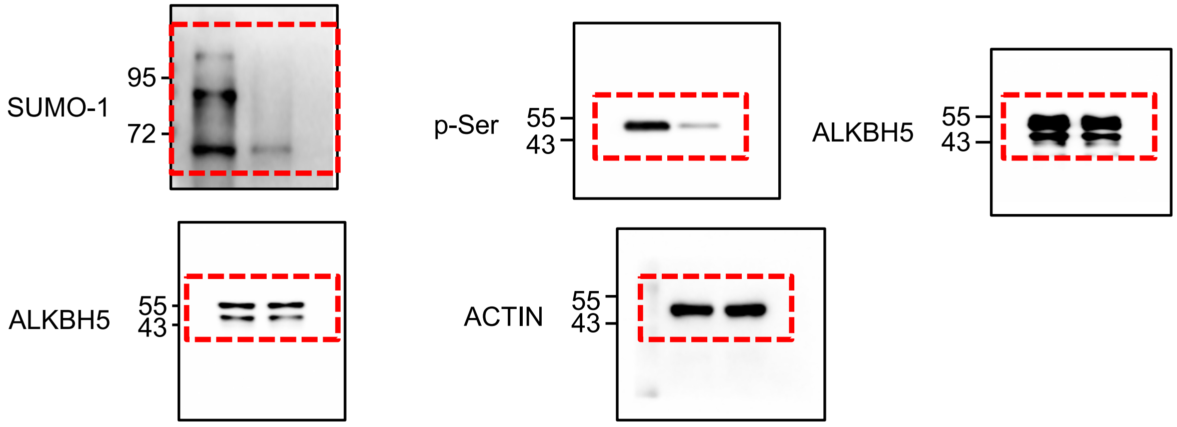

Full unedited Western blot. Red boxes indicate the images used in Supplemental Figure 3B.

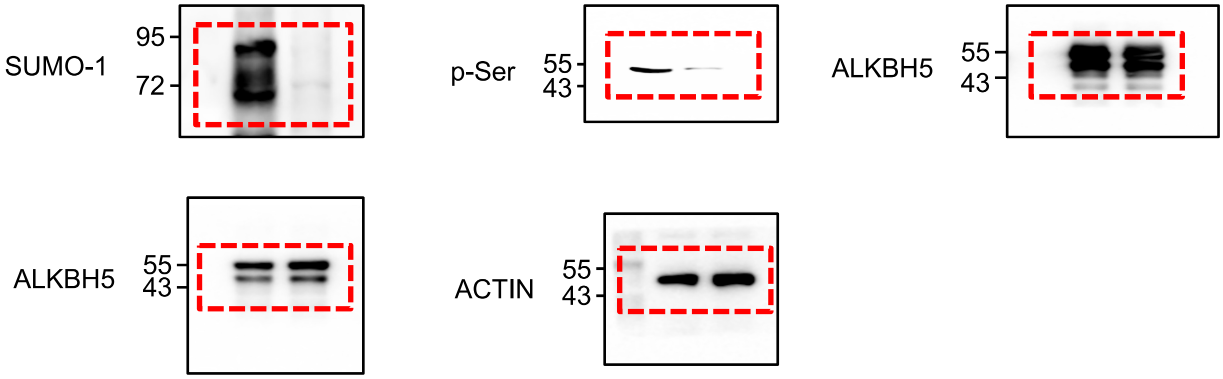

Full unedited Western blot. Red boxes indicate the images used in Supplemental Figure 3C.

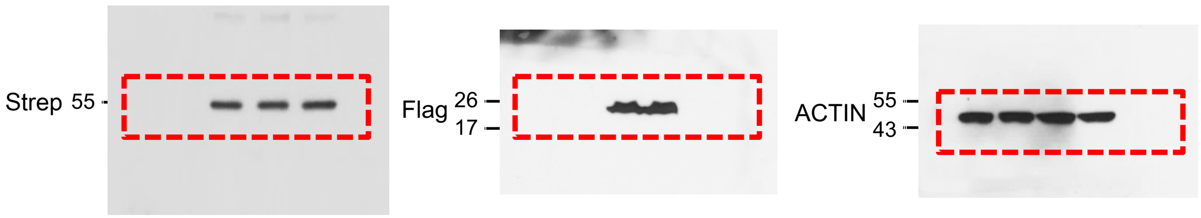

Full unedited Western blot. Red boxes indicate the images used in Supplemental Figure 3D.

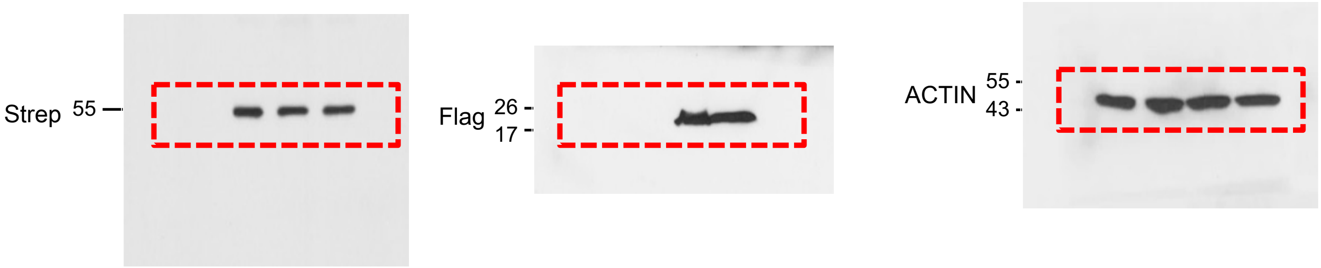

Full unedited Western blot. Red boxes indicate the images used in Supplemental Figure 3E.

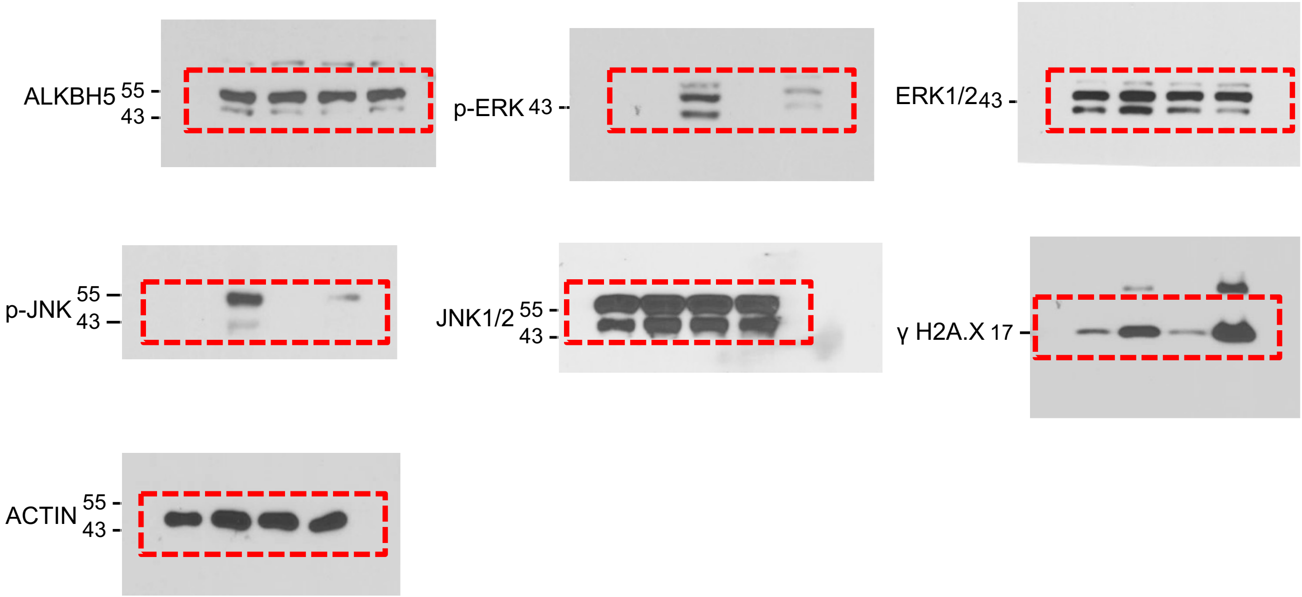

Full unedited Western blot. Red boxes indicate the images used in Supplemental Figure 3J.

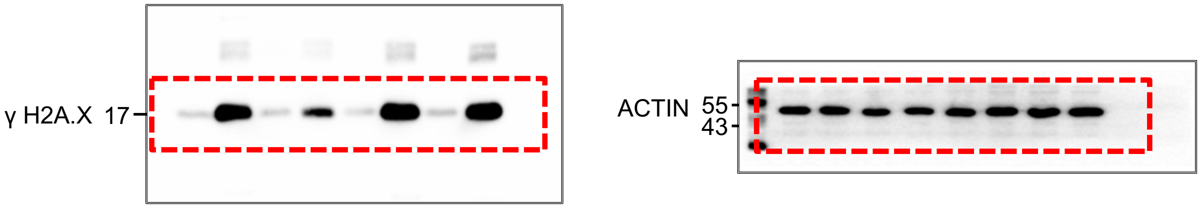

Full unedited Western blot. Red boxes indicate the images used in Supplemental Figure 6A.

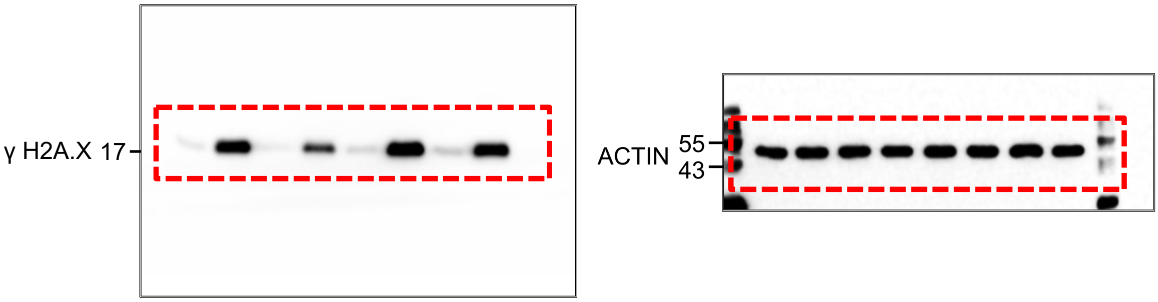

Full unedited Western blot. Red boxes indicate the images used in Supplemental Figure 6B.

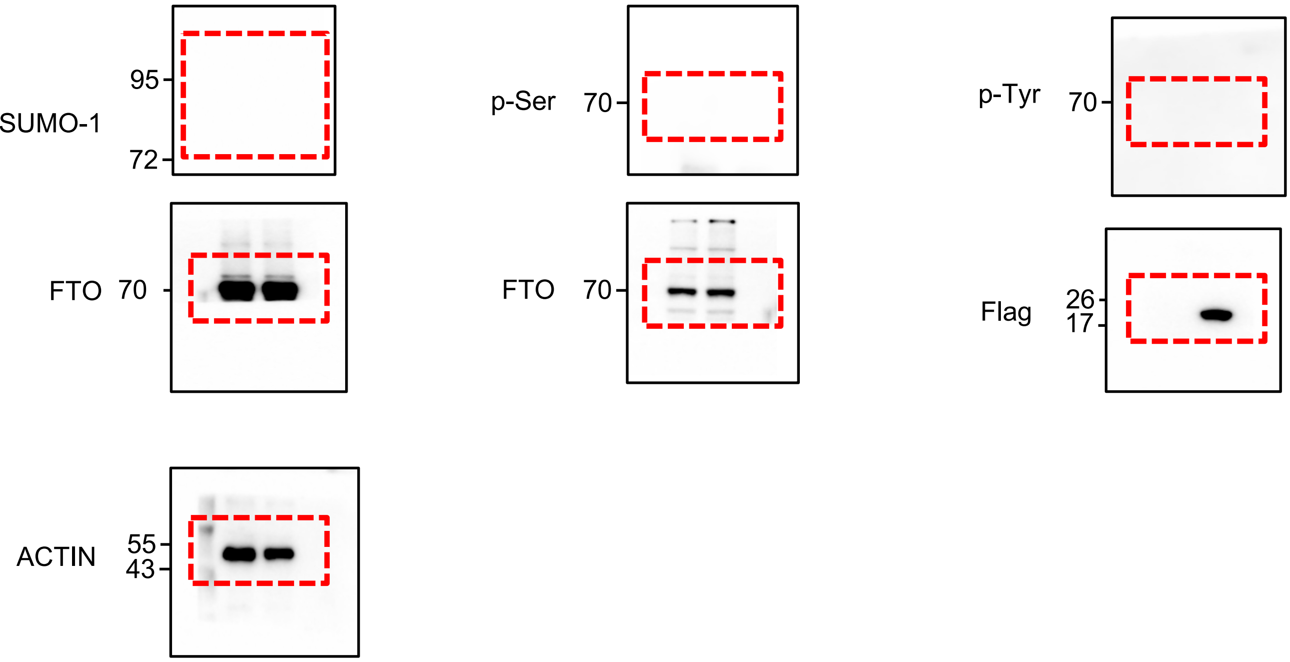

Full unedited Western blot. Red boxes indicate the images used in Supplemental Figure 7A.

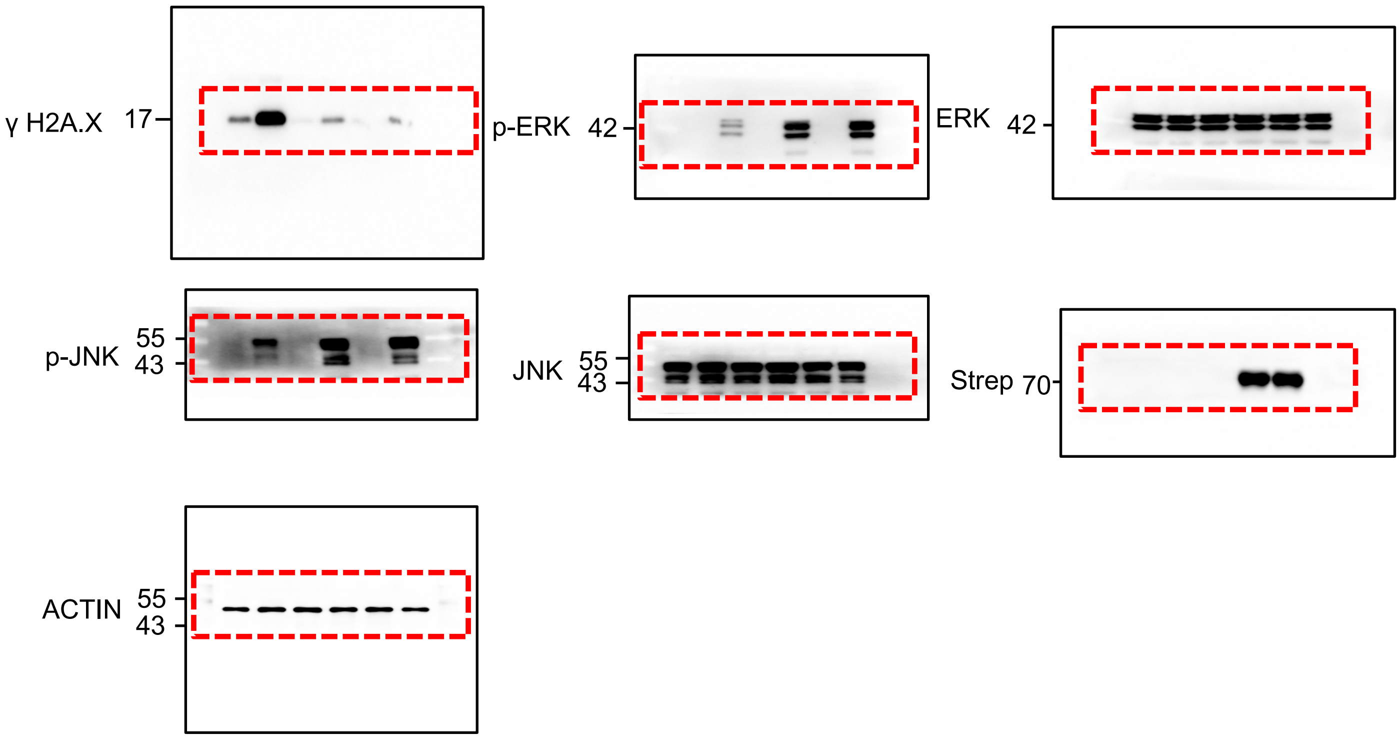

Full unedited Western blot. Red boxes indicate the images used in Supplemental Figure 7B.

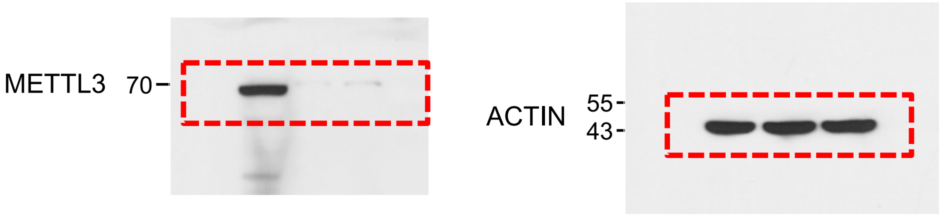

Full unedited Western blot. Red boxes indicate the images used in Supplemental Figure 8A.

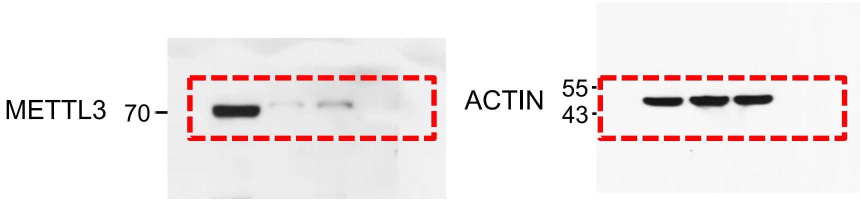

Full unedited Western blot. Red boxes indicate the images used in Supplemental Figure 8B.

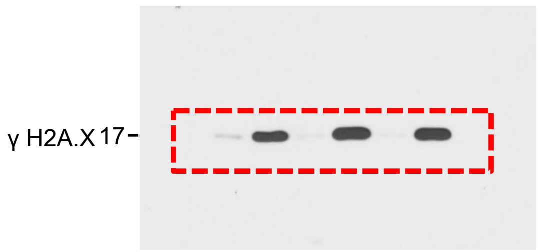

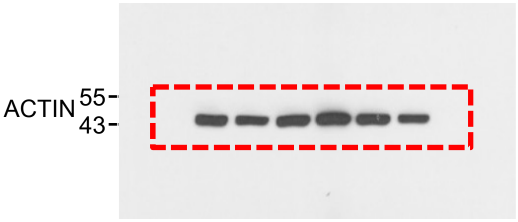

Full unedited Western blot. Red boxes indicate the images used in Supplemental Figure 8C.

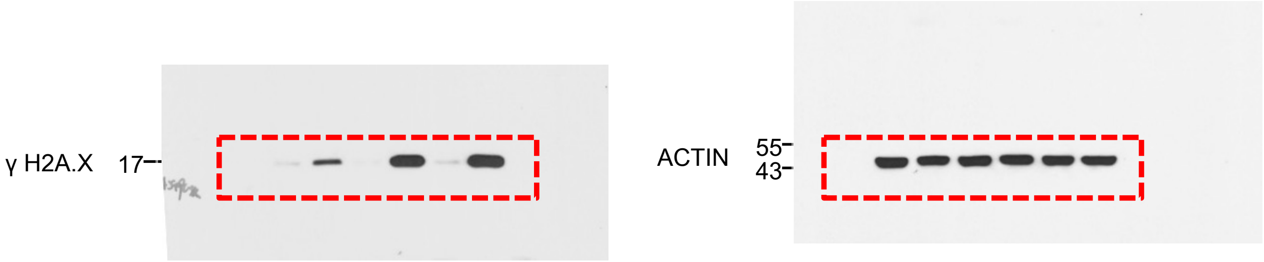

Full unedited Western blot. Red boxes indicate the images used in Supplemental Figure 8D.
